# Antibacterial activity of iron oxide, iron nitride, and tobramycin conjugated nanoparticles against *Pseudomonas aeruginosa* biofilms

**DOI:** 10.1186/s12951-020-0588-6

**Published:** 2020-02-18

**Authors:** Leisha M. Armijo, Stephen J. Wawrzyniec, Michael Kopciuch, Yekaterina I. Brandt, Antonio C. Rivera, Nathan J. Withers, Nathaniel C. Cook, Dale L. Huber, Todd C. Monson, Hugh D. C. Smyth, Marek Osiński

**Affiliations:** 1 Center for High Technology Materials, University of New Mexico, 1313 Goddard Street SE, Albuquerque, NM 87106-4343 USA; 2 Center for Integrated Nanotechnologies, Sandia National Laboratories, 1000 Eubank SE, Albuquerque, NM 87123 USA; 3 Sandia National Laboratories, Nanomaterials Sciences, P.O. Box 5800, MS 1415, Albuquerque, NM 87185 USA; 4 College of Pharmacy, The University of Texas at Austin, 2409 University Avenue, Stop A1900, Austin, TX 78712 USA

**Keywords:** Antibiotic resistance, *Pseudomonas aeruginosa*, Cystic fibrosis, Biofilm, Antibacterial agents, Drug delivery, Iron-oxide nanoparticles, Zero-valent iron nanoparticles, Magnetite, Alginate

## Abstract

**Background:**

Novel methods are necessary to reduce morbidity and mortality of patients suffering from infections with *Pseudomonas aeruginosa*. Being the most common infectious species of the *Pseudomonas* genus, *P. aeruginosa* is the primary Gram-negative etiology responsible for nosocomial infections. Due to the ubiquity and high adaptability of this species, an effective universal treatment method for *P. aeruginosa* infection still eludes investigators, despite the extensive research in this area.

**Results:**

We report bacterial inhibition by iron-oxide (nominally magnetite) nanoparticles (NPs) alone, having a mean hydrodynamic diameter of ~ 16 nm, as well as alginate-capped iron-oxide NPs. Alginate capping increased the average hydrodynamic diameter to ~ 230 nm. We also investigated alginate-capped iron-oxide NP-drug conjugates, with a practically unchanged hydrodynamic diameter of ~ 232 nm. Susceptibility and minimum inhibitory concentration (MIC) of the NPs, NP-tobramycin conjugates, and tobramycin alone were determined in the PAO1 bacterial colonies. Investigations into susceptibility using the disk diffusion method were done after 3 days of biofilm growth and after 60 days of growth. MIC of all compounds of interest was determined after 60-days of growth, to ensure thorough establishment of biofilm colonies.

**Conclusions:**

Positive inhibition is reported for uncapped and alginate-capped iron-oxide NPs, and the corresponding MICs are presented. We report zero susceptibility to iron-oxide NPs capped with polyethylene glycol, suggesting that the capping agent plays a major role in enabling bactericidal ability in of the nanocomposite. Our findings suggest that the alginate-coated nanocomposites investigated in this study have the potential to overcome the bacterial biofilm barrier. Magnetic field application increases the action, likely via enhanced diffusion of the iron-oxide NPs and NP-drug conjugates through mucin and alginate barriers, which are characteristic of cystic-fibrosis respiratory infections. We demonstrate that iron-oxide NPs coated with alginate, as well as alginate-coated magnetite–tobramycin conjugates inhibit *P. aeruginosa* growth and biofilm formation in established colonies. We have also determined that susceptibility to tobramycin decreases for longer culture times. However, susceptibility to the iron-oxide NP compounds did not demonstrate any comparable decrease with increasing culture time. These findings imply that iron-oxide NPs are promising lower-cost alternatives to silver NPs in antibacterial coatings, solutions, and drugs, as well as other applications in which microbial abolition or infestation prevention is sought.

## Background

The discovery of antibiotics in 1928 was undoubtedly one of the most important developments in medicine to date, responsible for saving millions of lives by making formerly deadly infections curable [1]. Antibiotic reliability is the foundation for modern medicine and has facilitated the development of numerous, formerly impossible, medical procedures. Virtually every aspect of what we call modern medicine: treatment of autoimmune diseases and allergies, therapeutic use of corticosteroids or other immunosuppressant drugs, chemo- and radiation therapy, any and all surgical procedures, burn and wound treatment, to include any procedures or accommodations in which stents, catheters, orthodontic wires, ventilators, staples, sutures, bandages, clamps, belts, implants, or virtually any procedure in which an inert object-biological interface exists; they all put the patient at risk for infection. The development of antibiotic drugs made all this possible. On the other hand, researchers and medical professionals alike continue to struggle with the intensifying issue of antibiotic resistance, especially prominent in healthcare environments, which threatens to collapse the crucial foundation on which modern medicine was built.

*Pseudomonas aeruginosa* is one of the notorious ESKAPE pathogens (a group consisting of *Enterococcus faecium*, *Staphylococcus aureus*, *Klebsiella pneumoniae*, *Acinetobacter baumannii*, *Pseudomonas aeruginosa*, and *Enterobacter* species), which have developed resistance to the bulk of our current antimicrobial regimes, and instead “escape” the lethal action of antibiotics [2]. More specifically, many highly resistant Gram-negative bacteria from the ESKAPE group, including *P. aeruginosa*, are emerging as exceptionally noteworthy pathogens in threatening public health in United States as well as other parts of the world [3]. The ESKAPE bacteria are of tremendous concern because they are responsible for causing the overwhelming majority of nosocomial infections. Several reports identify significant limitations in current treatment options for these pathogens that force medical professionals to settle on the use of previously discontinued drugs having documented toxicity and unclear dosage and administration guidelines [4–8]. They also provide complex models of pathogenesis, transmission, and drug resistance [2, 3]. Treatment regimens found to exhibit success against the ESKAPE bacteria can be applied to virtually any other species. Successful treatment of these species alone will result in significantly safer healthcare environments, more suitable for treating disease and illness.

*Pseudomonas aeruginosa* belongs to the Gram-negative Gammaproteobacteria class [9] and the Pseudomonadaceae family. Of all the species in the *Pseudomonas* genus, *P. aeruginosa* is the most common agent causing infections in humans [10]. It is abundant in the environment in general, and especially copious in the water and wastewater systems [10], making accidental inoculation difficult to avoid. *P. aeruginosa* infections are implicated in the morbidity and mortality of a wide spectrum of immunocompromised patients [11, 12]. The seriousness of the problem with multiple-drug resistant *P. aeruginosa* has been highlighted in a recent WHO report, which placed it in the highest global priority “critical” category, together with *A. baumannii* and Enterobacteriaceae [13]. In the United States, an estimated 51,000 healthcare-associated *P. aeruginosa* infections are reported each year [14]. Of these, more than 6000 (13%) are patients infected with multidrug-resistant strains, and approximately 400 deaths per year are attributed to these infections [14]. *P. aeruginosa* is not only one of the leading pathogens responsible for nosocomial infections [15–18], but also causes the morbidity and mortality of oncology and cystic fibrosis (CF) patients, where it is implicated in more than 90% of the occurrences of respiratory failure [19]. It is also prevalent in the burn units [20, 21], the intensive care units causing ventilator-associated infections [22, 23], and the neonatal intensive care units [24]. *P. aeruginosa* is the most prevalent isolate in intensive care units (ICUs), accounting for 23% of isolates, and the most common isolate taken from the human respiratory tract, accounting for 32% of isolates [25]. Ventilator-associated pneumonia (VAP) is a major cause of morbidity and mortality responsible for 25% of infections in ICUs [26, 27]. *P. aeruginosa*-related VAP results in high mortality, ranging from 40% to nearly 70% [28–31]. A recent report has also provided evidence that, of all the microbes causing bloodstream infections, *P. aeruginosa* is the one most commonly associated with mortality [32]. It possesses a significant number of virulence factors that work against the patient’s immune system, making the bacteria highly adaptable and often lethal [33].

Production of a biofilm also contributes to the ability of *P. aeruginosa* to elude antibiotic treatments and host immune defenses; phagocytosis is frustrated, and antibody penetration is limited [34]. The phenotypic switch to the biofilm mode of growth is governed by gene modulation [35]. Van der Waals forces initially hold planktonic bacterial cells to a surface, where they can then use appendages such as flagella, cilia, or pili as an anchor for stronger adhesion [34]. It has been demonstrated that, during the attachment phase, the genes encoding synthesis of the extracellular matrix are activated [34, 36]. The biofilm mode of growth is characterized, in general, by a reduced bacterial growth rate compared to the planktonic mode, the production of a protective extracellular polysaccharide (EPS) layer, and biofilm-specific gene activation [37]. The EPS layer constitutes a physical barrier, interfering with the ability of therapeutic antibiotic drugs to interact with the bacterial cells and exert their action. It is composed of several polymers as well as DNA; however, it consists primarily of alginate. Production of the alginate EPS by *P. aeruginosa* was initially discovered in 1966 by Linker and Jones [38]. Alginate is an anionic (negatively charged) co-block polymer composed of β-d-mannuronic acid (M) and C-5 epimer α-l-guluronic acid (G) residues attached in a linear fashion by 1–4 linkages [36, 39–42].

**Fig. 1:**
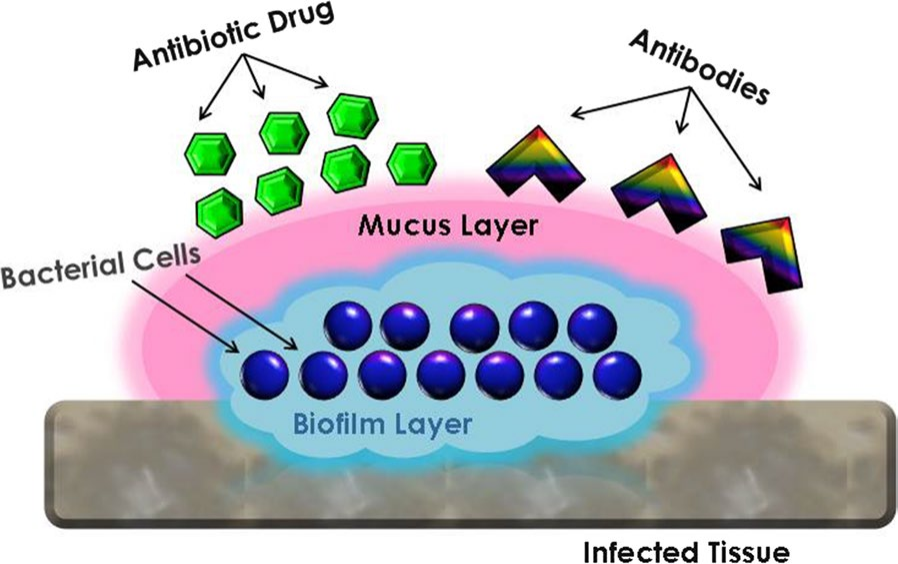
Biofilm colony on infected tissue with viscous mucus layer characteristic of an infection in the CF respiratory tract, illustrating the inability of drugs and antibodies to penetrate the mucus and biofilm barriers and reach the target microbial colonies

Biofilm infections are caused by multiple microbial species and, in general, are especially discommoding since they facilitate a means by which microbes are able to not only colonize a host tissue, but also inert objects such as surgical sutures, where they often cause chronic surgical site infections [43–45], orthodontic wires [46, 47], urinary or urethral catheters [48–50], venous or vascular catheters [51, 52], ureteral stents [53, 54], frontal recess stents [55], biliary stents [56] and respiratory (endotracheal) tubes [57, 58], among others. Biofilms also escalate the severity of infections in the respiratory tract, burns, and other open wounds, as well as virtually any other organ system [59]. The reduced efficacy of therapeutics caused by the presence of biofilm EPS layer serving as a barrier to antibiotic drug penetration limits treatment options even for antibiotic-susceptible strains [60]. In the case of treatment of biofilm infections in the CF respiratory tract, two barriers to drug diffusion exist: the biofilm EPS layer and the thick CF mucus layer (Fig. 1).

Significant research on silver NPs as antimicrobial agents has been reported in the literature [61–87] and much research has also been done on the efficacy of silver NPs against *P. aeruginosa* [88–99]. Due to their undisputable antibacterial properties, silver NPs are among the most commonly exploited nanomaterials in commercialized products [100]. Although silver NPs have demonstrated antimicrobial properties against many bacterial species, silver is costly, and is also known to exhibit toxicity in multiple species [101–103], including in vitro cytotoxicity in various human cell lines [100, 104, 105]. Most researchers attribute the observed toxicity either to silver ions [101] or the combination of silver NPs and silver ions [103, 104]. An ideal bactericidal agent should be lethal to bacteria, but safe to human cells. One such candidate is iron and its compounds. Iron-oxide NPs have been shown to be non-toxic [106–109]. For example, feraheme/ferumoxytol containing superparamagnetic iron-oxide NPs was approved by the U.S. Food and Drug Administration as an iron supplement for treatment of iron deficiency in patients with renal failure [110–112]. According to a previous report, iron-oxide in NP form is not only non-toxic, but its byproduct, degraded iron from the cores, apparently accumulates in natural iron stores in the body [113]. Properly biofunctionalized iron-oxide NPs have been shown to inhibit growth of *Staphylococcus aureus* [114–116] and *Escherichia coli* [115, 117], prevent biofilm formation by *P. aeruginosa* [118] and *Streptococcus mutans* [119], and exhibit bactericidal activity against a range of Gram-negative and Gram-positive bacterial species [120–125]. While these are very encouraging results, more work is necessary in the investigation of iron-oxide NPs as a feasible alternative to silver NPs in the treatment of bacterial infections and for biofilm disruption.

It has also been established that the bactericidal activity of Fe_3_O_4_ NPs strongly depends on the surface coating and should be individually optimized for maximum effect on each bacterial species of interest [115, 124]. For example, changing the zeta potential of as-synthesized iron-oxide NPs from negative to positive by coating their surface with biocompatible chitosan has significantly increased their antimicrobial activity against Gram-positive *Bacillus subtilis* and Gram-negative *Escherichia coli*, reducing the percentage of viable cells after 22-h exposure to 50 µM of NP suspension in nutrient broth from over 60% to less than 30% for either bacterial species [124]. Significant documented evidence suggests that the properties of the surface coating may account for the large differences in sensitivity results reported by various groups investigating the antibacterial effects of iron oxide, as the NPs used in the experiments often have different coatings. More research is necessary to thoroughly characterize the effects of surface coating on antibacterial properties.

In this paper, we report on the synthesis and characterization of iron-oxide (nominally magnetite) NPs capped with alginate and investigate the susceptibility of *P. aeruginosa* PAO1 bacterial colonies to iron-oxide NPs alone, as compared to tobramycin-conjugated iron-oxide NPs. Because uncapped iron-oxide NPs are unstable and aggregate at physiological pH of 7.4, they must be coated with a biocompatible polymer [126–128]. The coating must also contain additional, unbound functional groups for conjugation to a drug. For this study, we have opted to cap the NPs with natural alginate, rather than a synthetic polymer, to maintain green methodology and natural reagents. In addition, we anticipated that, since the bacterial biofilm is mostly composed of alginate, which is negatively-charged [129], the alginate-coating will impart a negative charge on iron-oxide NPs. It is well established that capping can be used to alter the surface charge of the iron-oxide NPs [125–128]. In our case, giving the nanocomposites similar negatively charged electrostatic properties to the target environment should promote diffusion through the alginate biofilms.

Iron-oxide (nominally magnetite) NPs with spherical morphology and a mean radius of ~ 16 nm were used for this study. The NPs were synthesized using a solvothermal method on a Schlenk line under inert gas flow. After synthesis, the NPs were capped with natural alginate and half of the batch was crosslinked to tobramycin using 1-ethyl-3-(3-dimethylaminopropyl)carbodiimide hydrochloride (EDC, also abbreviated as EDAC or EDIC)/N-hydroxysulfosuccinimide (sulfo-NHS). Characterization was done using transmission electron microscopy (TEM), dynamic light scattering (DLS), X-ray diffraction (XRD), and energy-dispersive X-ray spectroscopy (EDS). Tobramycin-conjugated NPs, as well as unconjugated NP samples were used to determine the susceptibilities and minimum inhibitory concentrations (MICs) of established *P. aeruginosa* colonies. All the microbiological procedures were carried out in a Biosafety Level 2 laboratory. The susceptibility to zero-valent iron NPs was also investigated as a positive control. Tobramycin antibiotic was chosen for these studies because it has been shown to be the most active drug tested on clinical isolates of *P. aeruginosa* and has exhibited excellent activity against multidrug resistant (MDR) strains, usually in combination with other antibiotics [130–142].

## Methods

### Synthesis and characterization of nanoparticles and nanoparticle-drug conjugates

For the synthesis of iron-oxide (magnetite) NPs, we have adopted a two-step procedure of Park et al. [143], with subsequent modifications reported in [144]. Step one is the synthesis of the organic carrier, which delivers the metal atoms individually to the growing lattice. Step two is the high-temperature growth of the nanocrystalline particles. The organic carrier was the coordination complex iron oleate, (iron(II, III) [(9Z)-9-octadecenoate] n), where n is the coordination number of iron. This molecule may form a monomer, dimer, or trimer [144, 145]. Iron oleate was produced in our laboratory using a procedure published in [144]. The iron oleate complex was synthesized in an ion-exchange reaction between sodium oleate salt (sodium (9Z)-9 octadecenoate) and iron(III) chloride hexahydrate (FeCl_3_·6H_2_O). In this reaction, the sodium atom on the sodium oleate is replaced by iron, forming iron oleate, and the chloride and sodium ions combine to form sodium chloride. The iron oleate synthesis procedure was modified by adding an additional washing step. The product was washed with deionized (DI) water, ethanol, and acetone, to remove additional contaminants and purify the product prior to aging in the oven overnight. After the iron oleate was aged overnight in the oven, the NPs were synthesized from it, using a high molecular weight inert hydrocarbon as the solvent, and oleic acid as a stabilizing agent. In this method, the time separation between nucleation and growth events can be maximized to achieve better monodispersity as well as morphology control [144–146]. Zero-valent iron NPs were synthesized using the same procedure with the addition of sodium borohydride to reduce iron oxide to iron under inert gas flow. NPs were then capped with either alginate or succinylated polyethylene glycol (PEG) 5000. Aliquots of alginate-capped iron-oxide NPs were then crosslinked to tobramycin via EDC/sulfo-NHS.

#### Materials

Iron(III) chloride hexahydrate (97%), sodium borohydride powder (98%), m-PEG 5000 (methyl-terminated PEG) powder, sodium alginate from brown algae (A2158), succinic anhydride (> 99%), phosphate-buffered saline (PBS) powder, and TRIS hydrochloride (PharmaGrade) were purchased from Sigma-Aldrich; hydrochloric acid solution 0.02 N was purchased from Fisher Chemical; *n*-docosane (99%) was purchased from Alfa Aesar; sodium oleate (> 97%) was purchased from Tokyo Chemical Industry Co.; hexanes (95%), ethanol (99%), acetone (99%), chloroform (99.9%), hexane (99%), anhydrous pyridine (99%), and methanol (99%) were purchased from EMD Chemicals Inc.; EDC hydrochloride (cat# 22981) and N-hydroxysulfosuccinimide (sulfo-NHS) (cat # 24510) were purchased from Thermo Fisher Scientific. All chemicals were used as received, without further purification.

#### Synthesis of iron oleate precursor complex

In a 1000-mL three-neck flask with a condenser, using standard air-free conditions, 25.92 g of iron(III) chloride hexahydrate (FeCl_3_·6H_2_O) and 87.60 g of sodium oleate were solvated by 96 mL of DI water, 192 mL of ethanol, and 336 mL of 99%-hexane (to dissolve the organics). Under argon flow, the flask was heated to 70 °C while stirring at 3000 rpm. The flask was kept refluxing at this temperature for 4 h. Then, the product was washed 3 times with 96 mL of DI water (in 32-mL aliquots) using a separatory funnel. The mixture was then placed in a rotary evaporator (RotoVap) to evaporate away any remaining hexane. The flask was then vacuum sealed, and placed in the oven for 24 h at 70 °C. The final product was a dark brown solid.

#### Synthesis of iron-oxide (magnetite) NPs

In a 500-mL three-neck flask with a condenser, 16.20 g of the iron oleate complex produced above was combined with 2.57 g of oleic acid. 30 mL of either paraffin wax or docosane was added to the flask as a solvent, along with 5–10 boiling stones, since a characteristic nucleation event occurs at 200 °C and may cause the pressure to increase significantly. Under argon flow, the mixture was heated to 370 °C at a rate of 5 °C per minute and held at that temperature for 15 min. After synthesis, the mixture was cooled to 50 °C, and NPs were washed three times with 95%-hexanes and acetone, and redispersed in chloroform. The iron-oxide NPs used in this study have a very narrow size distribution and the procedure produces consistent 16–17 ± 2 nm NPs per batch. The same batch of iron-oxide NPs characterized here was used as a precursor for the zero-valent iron NPs and iron nitride NPs, as well as in the biological studies.

#### Synthesis of zero-valent iron NPs

The NPs were synthesized in docosane in the same 500-mL three-neck flask using the same method as above. However, the reactants were reduced with a molar equivalent of sodium borohydride (NaBH_4_), as reported in the literature [147–151]. The reaction was carried out at 250 °C and kept at that temperature for 2 h. The NPs were separated from the docosane by diluting with a nonpolar solvent (95%-hexanes) and using a permanent magnet for magnetic separation under air-free conditions (in an atmosphere-controlled glove box), Subsequently, the NPs were annealed under air-free conditions to remove any remaining organics prior to being capped with alginate as described below.

#### Removal of oleic acid cap

The iron-oxide NPs came out of synthesis capped with the oleate via a carboxyl group bound to the metal atoms, since iron oleate served as the organometallic (metal carbonyl) complex by which the iron was delivered to the iron-oxide crystal [144–146]. The oleate cap was removed with a hydrochloric acid wash.

The process of removing the cap is governed by the Henderson-Hasselbalch equation [152, 153], which derives the pH as a measure of acidity from pK_a_ (the negative log of the dissociation constant) and the ratio of the concentrations of an undissociated acid and its conjugate base [154]:

1 $$pH = pK_{a} + log_{10} \left( {\frac{{\left[ {A - } \right]}}{{\left[ {HA} \right]}}} \right)$$

where [*A*^−^] is the conjugate base (oleate anion) concentration, and [*HA*] is the organic acid (oleic acid in our case) concentration.

The pK_a_ is given by [155]:

2 $$pK_{a} = - log_{10} \left(\frac{{\left[ {H_{3} O^{ + } } \right]\left[ {A^{ - } } \right]}}{{\left[ {HA} \right]}}\right)$$

where [H_3_O^+^] is the hydronium ion concentration.

When the pH is equal to the pK_a_, there will exist, in solution, an equal amount of protonated (acid) and deprotonated (conjugate base) molecules ([A^−^]/[HA] = 1. A typical carboxylic acid has a pK_a_ between 4 and 5 [154], however, titration experiments have shown that oleic acid has a much higher pK_a_ of 9.85 [155]. An organic acid will be significantly deprotonated in a solution if its pK_a_ is two or more units lower than the pH of the solution. Although the reaction would have proceeded at a higher pH, we used an HCl solution having a pH of 1 to ensure a more rapid protonation and thus, detachment of oleate from the iron-oxide NP at 25 °C. Inserting our pH value of 1 and the oleate pK_a_ of 9.85 into Eq. 1 returns a value of 6974.3 for the ratio [HA]/[A^−^]. Upon reaching this pH, the yellow-tinged transparent oleic acid could be visually observed to fall out of solution. The NPs were separated in a 95% hexanes/methanol mixture, in which the methanol solvated the oleic acid.

#### Alginate capping

Sodium alginate from brown algae is produced by the species *Macrocystis pyrifera* and consists of mannuronic and guluronic acids with an M/G ratio of approximately 3/2 and an average molecular weight of 46 kDa. Once the NP samples were uncapped and washed, sodium alginate was added to the NPs in a 1 to 3 alginate to NP ratio (by mass) in chloroform solvent. The mixture was sonicated at 40 kHz for 4 h to ensure complete coverage. The alginate-capped NPs were washed three times in chloroform using centrifugation and dried in air. The alginate-capped NP could be stored long-term in powder form or reconstituted in water for immediate use. This capping procedure is the same for zero-valent iron and iron-oxide NPs.

#### Polyethylene glycol succinylation and capping

Alternatively, instead of alginate, iron-oxide NPs were capped with a non-biodegradable polymer, succinylated PEG. To enhance the binding affinity of PEG-OH to the NPs, we further engineered mPEG using a simple succinylation procedure. mPEG-5000 was chosen, as it is an FDA-approved polymer and its use in biomedical applications has been well-documented [156–163]. Succinylated PEG was produced in-house from the PEG-OH terminal of mPEG in a process, during which the terminal hydroxyl group was converted, by a small chain extension, to a more electronegative carboxyl group. This enhances binding affinity, and thus promotes long-term colloidal stability even under increasing salinities.

**Fig. 2:**
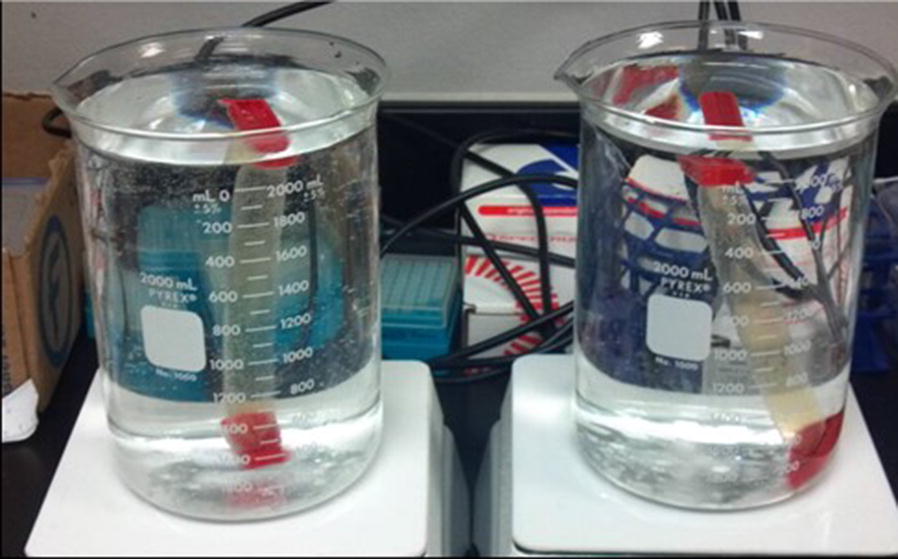
Purification of succinylated polyethylene glycol (PEG) through dialysis tubing in deionized (DI) water

For the reaction, five grams of 5000 molecular weight mPEG powder were added to a three-neck flask, purged with nitrogen, and placed on a stirring/heating mantle with a magnetic stir bar. Septa were placed on the other two flask necks, with a nitrogen adaptor on the central neck to purge with nitrogen. To keep a sealed pyridine bottle under close to atmospheric pressure, 25 mL of nitrogen gas were drawn up into a syringe through the septum of a nitrogen-filled three-neck flask connected to the Schlenk line and injected into the pyridine bottle. After injection, 25 mL of anhydrous pyridine were drawn up from the bottle and injected into the nitrogen-filled flask. The temperature controller was set to 50 °C, the temperature at which the solid mPEG dissolves. Subsequently, 2.5 g of succinic anhydride were added to the three-neck flask. This reaction process lasted for 1 h at 50 °C. The addition of pyridine was repeated four more times using the same methodology as described above, and the reaction could continue for another 2 h at 50 °C. The pyridine solvent, which has a boiling point of 115.3 °C at 1 atm, was removed using the rotary evaporator set at 40–50 °C to facilitate evaporation separation of the solvent. The evaporation procedure was followed by three DI water washes. The product was then re-dissolved in DI water and placed in 1-kDa-cutoff dialysis tubing in a 1-L beaker filled with DI water. The succinylated 5-kDa PEG was trapped inside, while the lighter precursor materials could diffuse out of the bag into the surrounding fluid (dialyte). The DI water in the 1-L beaker was replaced after 2, 4, and 8 h. Figure 2 shows the purification through dialysis with 1-kDa dialysis bags. After dialysis purification, the mixture was dried with the rotary evaporator, with the water bath set to 50 °C, the same as the synthesis temperature. The dried succinylated PEG was still liquid at this temperature and turned into a light-brown waxy solid when cooled to room temperature. After succinylation, PEG capping was performed using a modified procedure from [164]. For our work, we only used the succinylated PEG, prepared from mPEG as described above, as opposed to the costlier phospholipids with PEG tails used in [164].

The iron-oxide NPs were solvated in chloroform and combined with PEG using a NP-to-PEG mass ratio of 1:2. The NP/polymer solution was sonicated at 40 kHz for an hour at room temperature. The NPs were then washed three times with DI water via centrifugation, before being resuspended in DI water.

#### Conjugation to tobramycin

Drug conjugation to tobramycin was done using EDC with sulfo-NHS. Sulfo-NHS is a chemical modification reagent used in the conversion of carboxyl groups to amine-reactive esters in bioconjugation or crosslinking. Sulfo-NHS is a charged analog of NHS (N-hydroxysuccinimide) and, like NHS, facilitates control and alteration of carbodiimide crosslinking reactions in which carboxylates (–COOH), such as those present in the polymer molecule, are activated for conjugation with primary amines (–NH_2_) found on the tobramycin molecule. Such derivatives are synthesized by mixing the sulfo-NHS with a carboxyl-containing molecule, such as alginate, citrate, or carboxy-PEG, with a dehydrating agent such as the carbodiimide EDC. EDC is a “zero-length cross-linker”, meaning that it acts by bringing the two molecules of interest together, but does not change their combined size by increasing the polymer chain length. In the first step of the reaction, the carboxylated particles are activated by addition of the EDC, followed by the formation of a reactive ester intermediate, O-acylisourea. After that, the ester will react with an amine group forming an amide. However, this amide is highly unstable and will hydrolyze, regenerating the carboxyl group, if it does not encounter another amine functional group. Our procedure was modified for our purposes from a generic procedure for EDC crosslinking of carboxyl and amine terminal functional groups [165]. To conjugate the NPs capped with any of the above-mentioned organic molecules or polymers, 100 mg of Fe_3_O_4_ NPs washed three times with 10 mL of coupling buffer (50 mM phosphate buffered saline, pH 7.2) were removed by magnetic separation. The purified NPs were then suspended in 5 mL of coupling buffer. To ensure an excess of the ligand, 50 mg of tobramycin (50 mg of tobramycin per 100 mg of NPs) were dissolved in coupling buffer, thus making a 10 mg/mL tobramycin solution. Under gentle stirring, the NP solution was added drop-wise into a beaker containing the tobramycin solution, and allowed to sit for 2 min at 450 rpm. 100 mg of EDC for each 100 mg of NPs were added to the reaction mixture, under stirring until solvated. Then, 5 mM sulfo-NHS was also added to the reaction vessel. The conjugation reaction could proceed for 4 h at room temperature under gentle stirring. Afterwards, the NPs were washed twice with 5 mL of coupling buffer, before being resuspended in coupling buffer containing 35 mM Tris to block excess reactive sites. Finally, the NPs were washed twice again, suspended in DI water, and stored in the refrigerator. Conjugation to tobramycin was confirmed with Fourier-transform infrared (FTIR) spectroscopy done on EDC-crosslinked NP-tobramycin nanocomposites in KBr pellets.

### Nanoparticle characterization

#### Structural and compositional characterization

High-resolution TEM (HRTEM) measurements were taken with a JEOL-2010F transmission electron microscope operating at 200 kV and equipped with an Oxford Instruments 200 EDS apparatus, fitted with an Inca X-Site Ultra-Thin Window EDS detector. TEM samples were prepared by placing a drop of the colloidal solution onto a 200-mesh carbon-coated copper grid, and the solvent was allowed to dry, fixing the nanocrystals on the grid. To obtain elemental composition using EDS, the electron beam was focused on a single nanocrystal and the peaks were identified using the Oxford Instruments ISIS software. Data obtained from multiple single-nanocrystal measurements showed good repeatability. X-ray diffraction (XRD) was performed on a Rigaku^®^ Ultima III X-ray diffractometer. The colloidal sample was applied to a heated glass slide and the solvent was evaporated away, affixing the sample to the slide.

#### Size determination

Hydrodynamic size distributions of the nanocrystals have been measured using a DynaPro Titan DLS module from Wyatt Technology Corporation. In order to reduce aggregation and maximize the accuracy of the measurement, samples were prepared for analysis by diluting the NP@oleate stock solution to 50 µg/mL in pure chloroform. The NP@alginate stock solution was diluted in DI H_2_O. The 1-mL samples were vortexed, then sonicated at 40 kHz for 5 min prior to analysis to separate agglomerates and ensure that a more homogeneous solution was analyzed.

#### Magnetic characterization

Magnetic characterization was performed on oleate-coated NPs using a Quantum Design magnetic property measurement system (MPMS) superconducting quantum- interference device (SQUID) magnetometer. Magnetic hysteresis measurements were conducted at 293 K. Temperature-dependent magnetic properties were measured at a range from 10 to 350 K. To measure the zero-field cooling (ZFC) and field-cooling (FC) magnetization curves, three steps were carried out as follows: First, NP samples were gradually cooled in a zero magnetic field from room temperature to 5 K. Then, a magnetic field of 100 Oe was applied to measure the ZFC magnetization curve in a warming process from 10 to 350 K. Last, the FC curve was measured under the same applied field in a cooling process from 350 to 10 K.

#### Zeta potential measurements

Zeta potential measurements have been used to characterize the electrostatic potential at the electrical double layer that forms at the interface of a colloidal NP and the dispersing solvent. Although the zeta potential measurement is often regarded as NP surface charge, it is not actually a measure of surface charge. Zeta potential measures the potential difference between the dispersion medium and the adsorbed layer of solvent ions surrounding the particle. This is not equal to the surface charge or the Stern potential [166], which are defined at a different location. Colloids with a zeta potential between -10 and +10 mV are considered neutral, while colloids with a zeta potential greater than 30 mV or smaller than -30 mV are considered strongly cationic, or anionic, respectively [167]. Particles with a large measured value of zeta potential, whether negative or positive, are electrostatically stabilized, whereas particles with low absolute values of zeta potential aggregate or flocculate [167–169]. According to Liao et al. [170], iron-oxide NPs in water had a zeta potential of +16.1 mV (incipient stability), which shifted to − 60.1 mV (good–excellent stability) after capping with alginate. Because most cell membranes are negatively charged, zeta potential is a key parameter in membrane permeability, and cationic particles tend to exhibit toxicity associated with membrane disruption (lysis) [167]. In our case, the alginate coating will impart the nanocomposites similar negatively charged electrostatic properties to the target membrane and biofilm environment, which should promote diffusion through the alginate biofilms, while also imparting the colloid significant stability at physiological pH.

#### Fourier transform infrared (FTIR) spectroscopy

FTIR spectroscopy was performed on tobramycin-conjugated NPs to confirm the successful conjugation of the drug. Since neither the tobramycin molecule, nor the capping polymer have an amide linkage preexisting in their structure, the presence of an amide bond (1630-1681 cm^−1^) can be used to verify a successful EDC conjugation. The samples were dispersed in KBr pellets for FTIR analysis.

## Microbiological methods

*P. aeruginosa* cultures in 75% glycerol were preserved frozen in a liquid nitrogen tank. The broth medium was inoculated ~ 48 h prior to the start of the study. The liquid cultures were grown overnight on a rotary shaker at 37 °C and 150 rpm. After that, the cultures were diluted to an optical density (OD) at 600 nm (OD_600_) between 0.5 and 0.6. The cultures grown overnight in liquid media were tested for MIC of tobramycin, prior to being maintained as biofilm colonies. OD_600_ is a well-established method to determine bacterial cell concentration (in mg/mL) from the linear determination of colony forming units (CFUs), or viable cells capable of replication under the controlled conditions, in the media [171]. The number of CFUs corresponding to the optical density for *P. aeruginosa* at an OD_600_ = 1.0 is 2.04  × 10^8^ CFU/mL, which is equal to a bacterial concentration of 2.085 mg/mL [171]. OD_600_ was determined using Cary 5000 UV–VIS–IR spectrophotometer against a blank cuvette. This concentration was then used for subsequent inoculation of cultures used in the study.

*P. aeruginosa* PAO1 biofilm communities were grown on sterile boiling stones in liquid growth media for 60 days until firmly established. We have chosen PAO1, a derivative of the original Australian PAO isolate, as it is the most commonly used strain for research on this ubiquitous and metabolically versatile opportunistic pathogen. It has been distributed worldwide to numerous laboratories and strain collections. All MIC and susceptibility experiments were done in triplicate and repeated, for a total n of 6. MIC of tobramycin was measured over time on day 1 after one overnight incubation (in liquid culture without boiling stones), day 3, day 10, and day 60. Motility testing was done on cultures after 1-day, 3-days and 60-days of growth. Motility testing was done by preparing agar in test tubes and inoculating the agar using the stab technique with a sterile inoculation loop having a pointed end. In this method the sharp end of the inoculation loop is dipped into the cultures and stabbed into the agar inside of the test tube one time. The tubes are then incubated overnight and observed the next day. Motile strains can be seen to have disrupted the agar surrounding the place where the stab inoculation was inserted into the agar. This disruption of the agar is not detectable in non-motile strains.

**Fig. 3:**
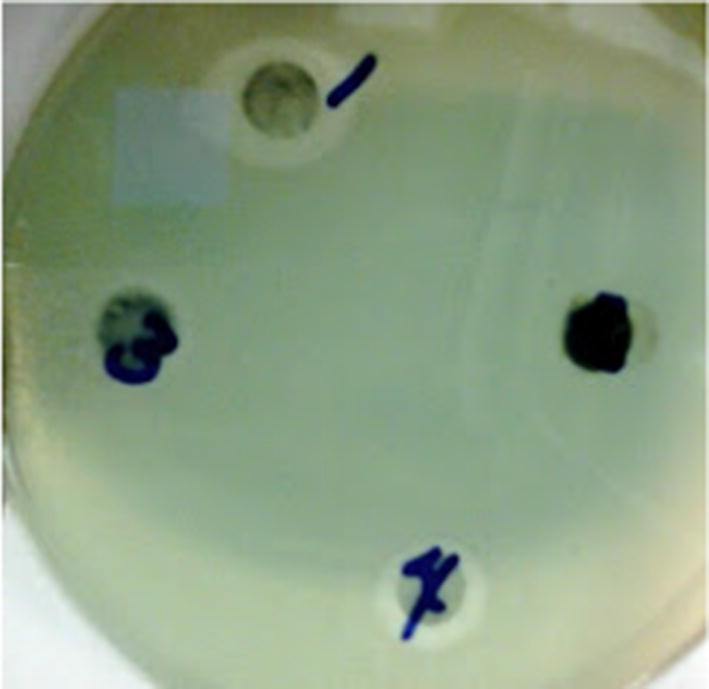
Image showing the presence of pyocyanin (blue-green) pigment produced by *P. aeruginosa* cultures grown on agar plates

Previous studies on *P. aeruginosa* biofilms reported using a growth period of 6 days [172, 173]. However, another group reported that after 7 days of biofilm formation the accumulation of biomass had not yet reached a plateau [174], while a classic publication reported that 5 weeks of growth was the optimal amount of time to achieve the maximum amount of biomass [175]. In yet another report, in which the mucoid phenotype observed in *P. aeruginosa* typical of CF infections was investigated, biofilm cultures were maintained for 90 days [176]. It has also been reported that 30 to 60-day-old biofilms are more resistant to some stresses [177]. Therefore, although much of research on *P. aeruginosa* is reported on biofilms which have undergone shorter term growth, it appears that short term cultures are only merited for in vitro diagnostics, as they were originally intended. In diagnostic studies, colonies are allowed to differentiate just long enough to obtain diagnostic and sensitivity information. Longer term studies, although costlier, are no doubt merited in research settings due to the documented difference and robustness of established colonies. Since we are interested in modeling a typical *P. aeruginosa* infection in CF patients which is an established infection, known to have more inherent resistance to antibacterial agents, we maintained our biofilms for a period of 60-days prior to testing susceptibility to NPs and NP-tobramycin conjugates. The established biofilm colonies produced a thick EPS matrix and several pigments characteristic of *P. aeruginosa* biofilms: pyocyanin (blue-green) (Fig. 3), pyoverdine (fluorescent yellow-green), and pyorubin (red-brown). The optical color changes were noted and crystal violet staining was done to verify the presence of biofilms.

Luria Bertani (LB) broth (cat #11006-004) and LB agar (cat #11006-001) were purchased from IPM Scientific, Inc. Eldersburg, Maryland, USA. Crystal violet staining was used to determine the amount of incubation time needed for biofilm formation. 100-µL dilutions of the *P. aeruginosa* (ATCC 27853) cultures were incubated overnight at 37 °C in LB broth in microtubes, without the addition of biofilm-promoting medium. Then, the liquid medium was decanted, and the tubes were gently rinsed with DI water. A 0.1% solution of crystal violet was prepared and ~ 100 µL of the solution was added to the microtube and incubated for 20 min before being rinsed 3 × with DI water. The presence of the biofilm appears as a “ring” in the tube, at the interface of the growth medium and air. After 48 h of growth biofilms were apparent, as mucoid colonies adhered heavily to the cap and were visible, having a sticky, “egg white” consistency, stretching from the cap to the vial whenever the cap was removed.

*P. aeruginosa* cultures were grown in LB broth on sterile boiling stones at 37 °C for 60 days. The liquid medium was decanted every 24–48 h, leaving only attached cells in the culture. The cells were then replenished with fresh broth. This method is a low-cost alternative to a flow chamber. After the 60-day period, the cultures were sonicated for the removal of attached cells, and once again, diluted to an optical density at a 600 nm wavelength (OD_600_) between 0.5 and 0.6. OD_600_ was determined using Cary 5000 UV–VIS–IR spectrophotometer against a blank cuvette, which contained only uninoculated broth. Once diluted, the cultures were tested in liquid media or applied to agar plates for susceptibility testing.

### Antimicrobial susceptibility tests

The disk diffusion method is one of the most popular approaches to bacterial sensitivity testing due to its low cost and efficiency. The disk, impregnated with a candidate antibiotic drug or compound of interest, was placed on the inoculated agar, which contained a uniform layer of bacteria taken from liquid culture. The disks are commercially available, containing the proper concentrations of antibiotic drugs recommended for susceptibility testing by the Clinical and Laboratory Standards Institute (CLSI), the institution responsible for maintaining standards for such research. The underside of the plate was numbered for each sample to be tested. Alternatively, disks containing different concentrations of the compound of interest can be prepared in the laboratory. The method used was the agar disk diffusion, as described by CLSI, with impregnated disks applied to the cultured agar plates overnight for 16–18 h [178].

Approximately 10^8^ CFU/mL of bacterial cultures, corresponding to ~ 1 mg/mL concentration, as determined by OD measurements, was distributed evenly onto a sterile agar plate using a sterile cotton swab to form a uniform layer on the agar. The disks, impregnated with NPs, drug, or NP-drug conjugates were then placed on top of the agar. A previous method of impregnation, the dip method, in which dry disks were dipped into known concentrations by forceps and then placed onto the agar cultures was found to produce inconsistent results because it was shown that the disks can absorb different amounts of liquid, introducing variability in the absorbed concentrations [179]. Instead, the more accurate drop method described by Sabath [180] was used. In this method, the dry disks are placed on the agar plates, then a known volume. The dry disks were placed atop the cultures and a 0.1 μL drop of the solution of interest at the desired concentration was applied to the disk using a micropipette calibrated micropipette. This method eliminates variability in the total absorbed amount since a known volume is applied. Disk concentrations of tobramycin were initiated at the CLSI recommended disk content for tobramycin, corresponding to 10 μg absorbed into the disk, when this mass returned a negative susceptibility, the concentrations were increased incrementally, until a susceptible mass was determined. For the initial disk diffusion study investigating tobramycin, NPs, and NP-conjugates, the mass on the disk was determined from concentration and applied volume. For example, a 0.1 μL aliquot of a solution having a concentration of 100 mg/mL corresponds to 10 μg in the disk (0.1 μL*100 mg/1 mL = 10 μg), a 50 mg/mL concentration corresponds to 5 μg in the disk (0.1 μL*100 mg/1 mL = 5 μg), and a 25 mg/mL concentration corresponds to 2.5 μg in the disk (0.1 μL*25 mg/1 mL = 2.5 μg), and so on. All doses based on concentration or weight correspond to the total nanocomposite, not to NPs, PEG, or tobramycin alone. The cultures were grown under the previous conditions overnight (16–18 h) at 37 °C. The diameter of zone of inhibition around the disc was observed and recorded. A CF biofilm, mucus model was also investigated on the 60-day-old biofilms, to determine if magnetic gradient guided transport enhanced susceptibility. For this model, the cultures were prepared on agar as described above, however, 1 mL of either prepared pig mucin, aqueous alginate, or both were applied on top of the plated colonies. The drug or NP-drug impregnated disks were applied atop the barriers. Half of the agar plates were placed on top of a ring magnet composed of sintered neodymium, iron, and boron magnetic alloy blendgrade N45, having a Gauss rating of 13,500 Gauss, a pulling force of 282 lbs, an axial pole orientation, a NiCuNi coating, and a tolerance of ± 0.002 inches. The magnets were placed below the agar plate for the entire overnight incubation.

### Determination of minimum inhibitory concentration determination

**Fig. 4:**
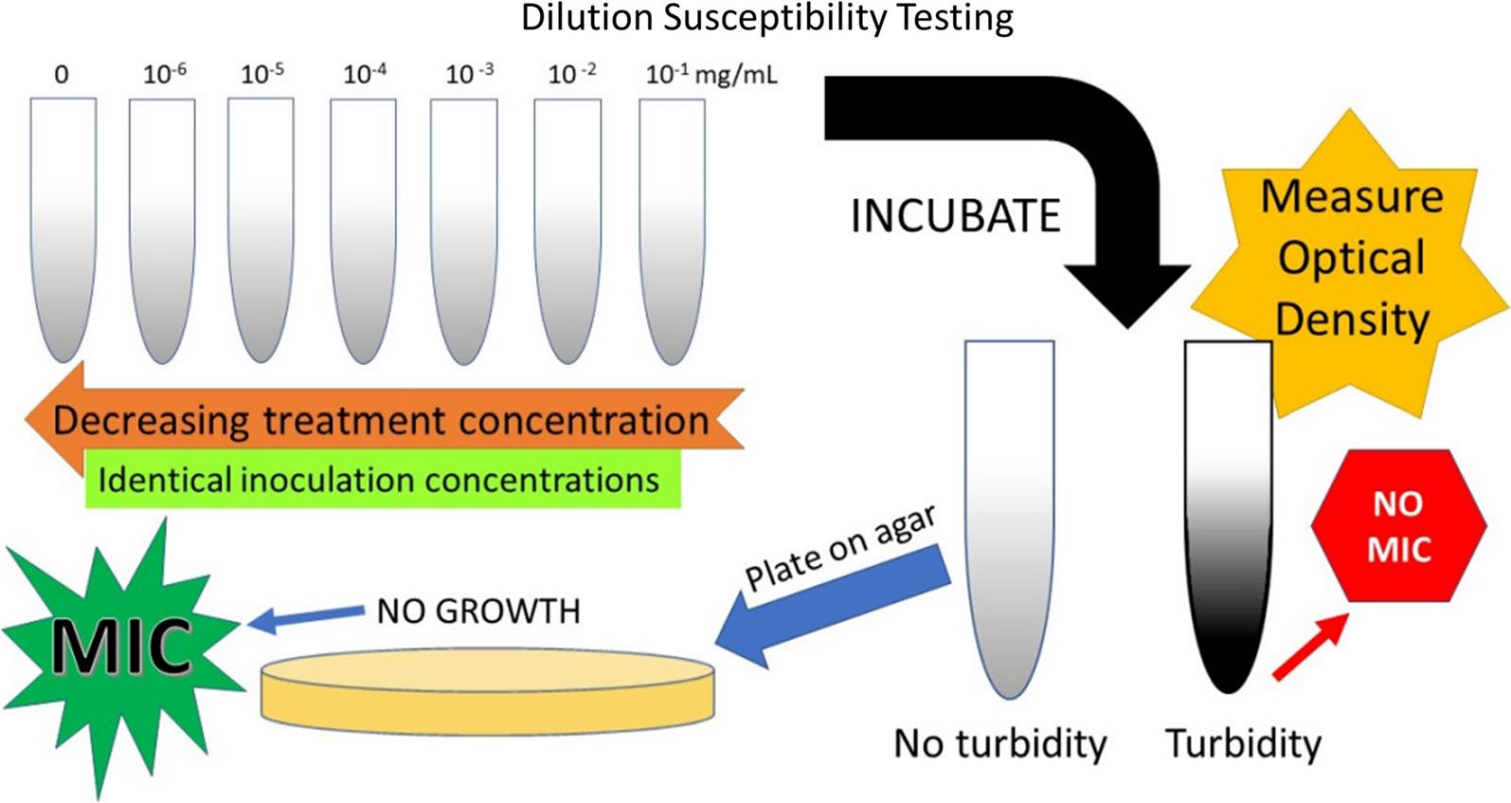
Schematic diagram of minimum inhibitory concentration (MIC) determination of tobramycin, iron-oxide NPs, tobramycin-NP conjugates, and zero-valent iron NPs in *P. aeruginosa* liquid cultures

For the MIC measurements, the compounds of interest (NPs, tobramycin, or NP-conjugates) were serially diluted in liquid growth media, inoculated from cultures grown for a specific period and incubated in sterile 2 mL vials overnight. The cultures were then grown overnight on a rotary shaker at 37 °C and 150 rpm. Optical density (OD) of liquid cultures was compared to a control cuvette containing only growth media, and ODs comparable to the growth media alone were considered inhibited growth. OD typically increased with decreasing treatment concentrations, as the bacterial cells were increasingly able to differentiate at the decreasing treatment concentrations. The MIC was narrowed down by using the dilution series with even smaller increments of tobramycin concentration, ranging between its highest concentration that still allowed the growth of *P. aeruginosa* colonies and the next lowest concentration that completely inhibited their growth. The MIC measurements are schematically illustrated in Fig. 4.

To verify inhibition, an inoculation loop was used to plate samples from liquid cultures, having been incubated overnight with a known treatment concentration, and having an OD comparable to growth media alone. These agar plates were allowed to grow overnight at 37 °C. MIC was determined by complete inhibition, defined by negative growth on agar as well as no apparent growth in liquid cultures determined by OD. For the control, sterile DI water was added to the aliquot of the culture, as opposed to an investigational compound. Due to the potential for interference of NPs with OD measurements, NPs were removed from solution, by magnetic separation, after inoculates were plated on agar, but prior to OD measurement.

### Comparison of biofilm inhibition in liquid cultures

OD was used to determine the number of cells in each liquid culture. The biofilms were sonicated to detach them from the boiling stones prior to being transferred to cuvettes for the OD_600_ (optical density at a 600 nm wavelength) measurements. As before, the measurements were taken against a blank cuvette containing only growth media.

### Graphical and statistical analysis

Graphical and statistical analyses analysis of variance (ANOVA) were performed on Microsoft Excel and GraphPad Prizm™. Average values and standard deviations were calculated on Microsoft Excel^®^ and ANOVA was performed on GraphPad Prizm™.

## Results

### Nanoparticle characterization

#### Transmission electron microscopy

**Fig. 5:**
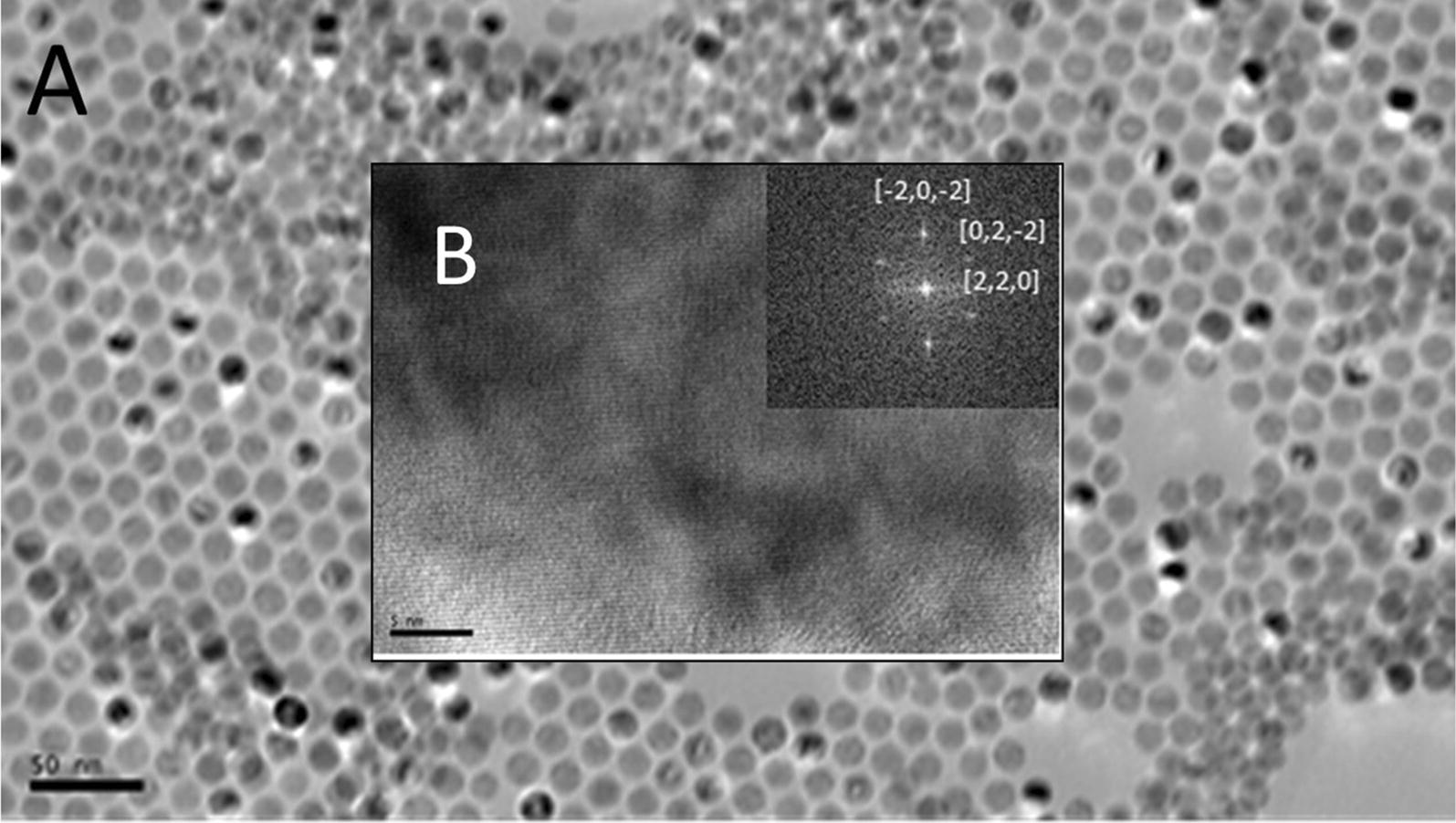
TEM images of iron-oxide NPs prior to alginate capping. Image A, scale bar is 50 nm, image B (insert) is a HRTEM image of crystal structure, scale bar 5 nm, and FFT of fringing with crystal indices

**Fig. 6:**
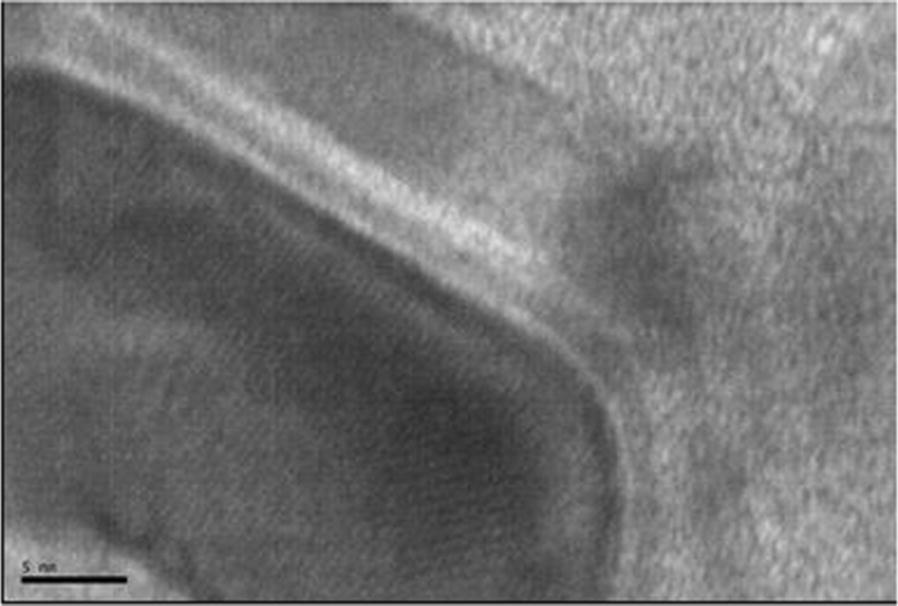
HRTEM image of an iron nitride NP showing crystalline fringes, scale bar is 5 nm

TEM results (Fig. 5) show a spherical morphology, high monodispersity, and the iron cores measure approximately 16 nm in diameter, in agreement with the DLS results taken for NPs coated with oleate. Figure 6 shows a TEM image of an iron nitride NP, showing an oblong, rounded-rectangular morphology. The iron nitride NPs were approximately 15-45 nm × 30-65 nm in size. It is important to note that the TEM analysis was difficult due to the strong magnetic interaction between the material and the electron beam. The strong magnetic properties of the sample caused the beam to oscillate, interfering with the analysis. Both the XRD and TEM show a body-centered tetragonal (BCT) crystal system. This system would be expected for Fe_16_N_2_, differentiating it from iron or iron oxide.

#### Energy-dispersive X-ray spectroscopy

**Fig. 7:**
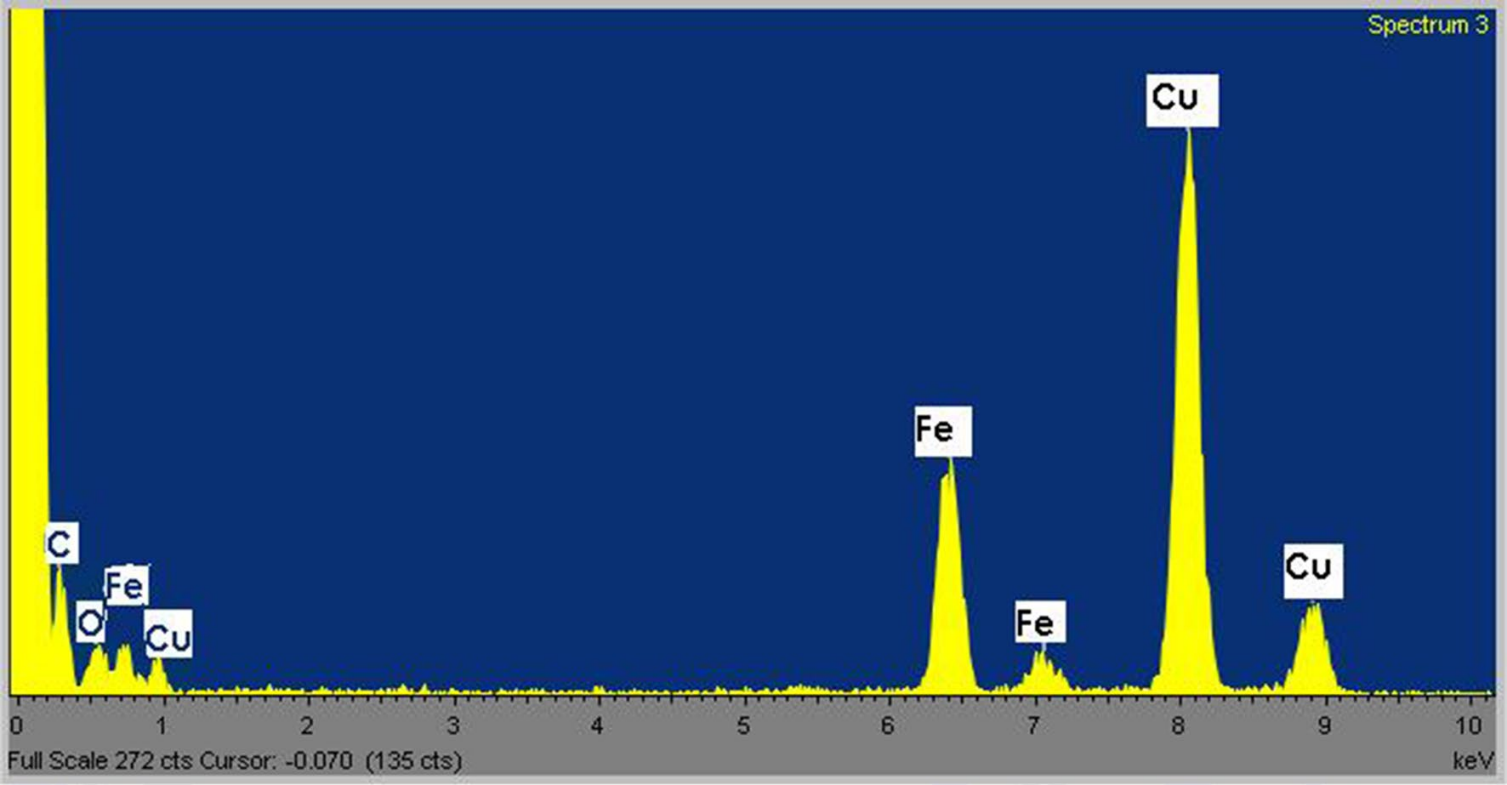
Energy dispersive x-ray spectrum of iron-oxide NPs, showing elemental composition

Elemental composition of the Fe_3_O_4_ NPs was verified with EDS and is shown in Fig. 7. EDS confirms the presence of iron and oxygen in the nanocrystal structure. The carbon and copper peaks are from the carbon-coated copper grid.

#### X-ray diffraction

**Fig. 8:**
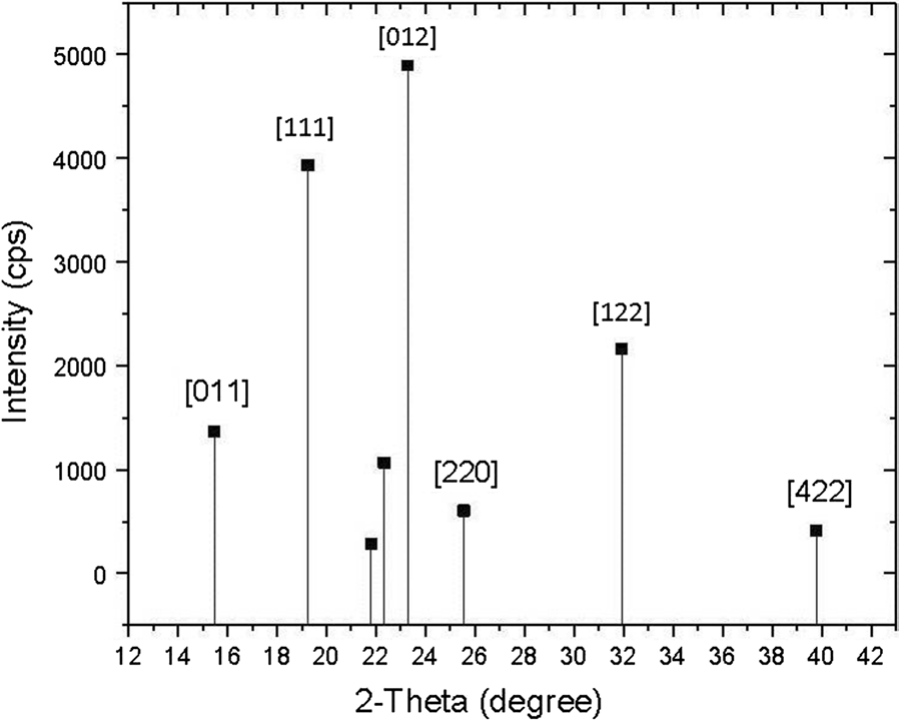
Indexed magnetite peaks from powder x-ray diffraction pattern obtained with a Cu Kα 1.54 Å source and a monochromator

**Fig. 9:**
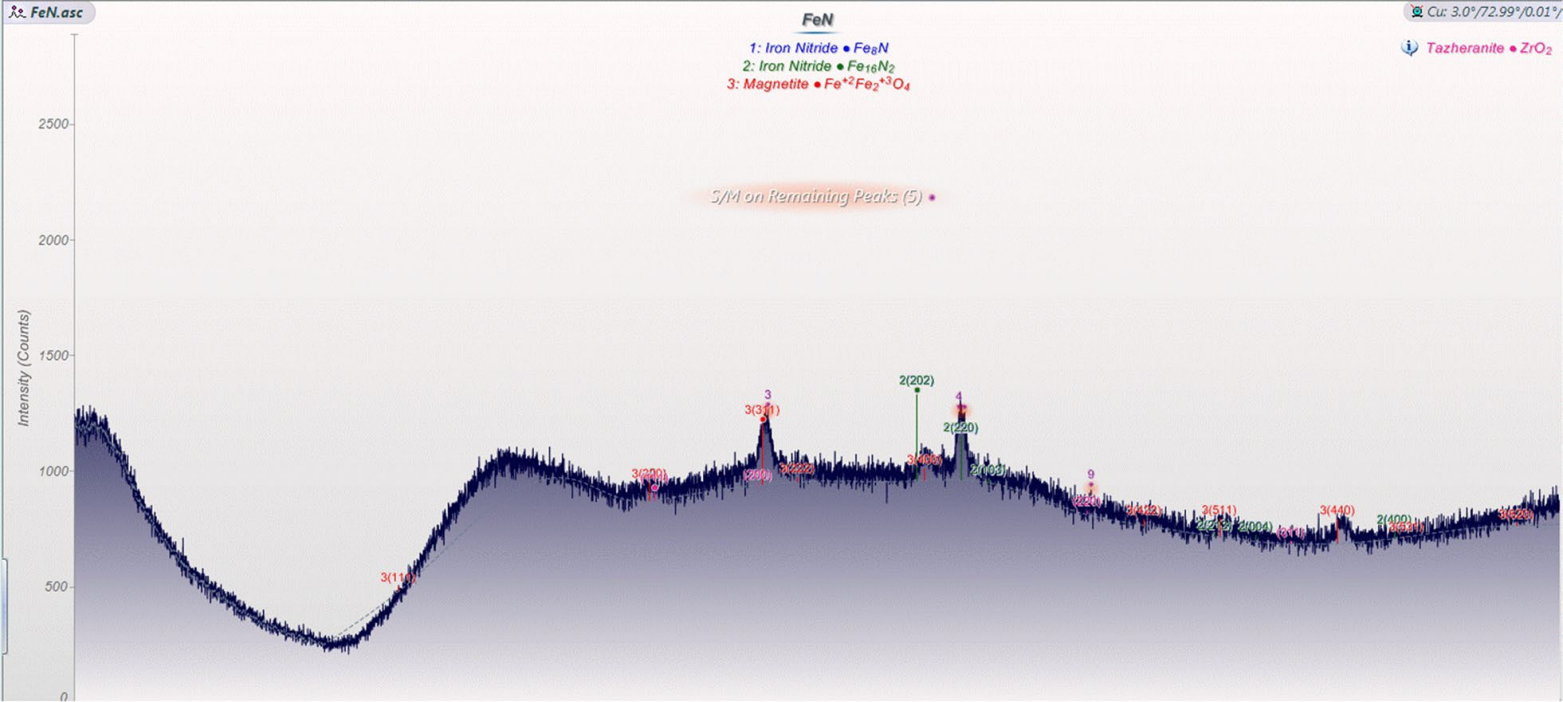
XRD spectrum of iron nitride NPs taken with a Cu Kα 1.54 Å source and a monochromator

The XRD data for iron-oxide NPs (Fig. 8) suggest that the composition of the NPs is 70–80% spinel-phase iron oxide, which we attribute to magnetite. The remaining 20–30% appears to be FeO (wüstite) and α-Fe_2_O_3_ (hematite), likely the result of surface oxidation of the alpha iron. It is important to note that due to the similarity in space groups and lattice constants as well as significant peak-overlap among the iron-oxide phases, the oxidation state of iron-oxide, and thus differentiation between phases, are difficult to determine with absolute certainty using XRD. On XRD analysis, zero-valent iron shows a single strong peak at ~ 44° (not shown). XRD spectrum for the uncapped iron nitride NP sample can be seen in Fig. 9. The Jade software automatched the spectrum to the iron nitride (martensite) phases α’-Fe_8_N, ICDD/ICSD card number 01-070-6150 and α’’-Fe_16_N_2_, ICDD/ICSD card number 01-078-1865, both tetragonal crystals with lattice constants *a* = 5.71 Å, *b* = 5.71 Å, *c* = 6.016 Å and *a* = 5.72 Å, *b* = 5.72 Å, *c* = 6.29 Å, respectively. The scan also reveals some magnetite (Fe + 2Fe_2_ + 3O_4_), ICDD/ICSD card number 00-019-0629, which is a cubic crystal with lattice constants *a* = 8.38 Å, *b* = 8.38 Å, *c* = 8.38 Å. This iron oxide likely resulted from surface oxidation of the uncapped NP sample which was set onto the slide using ethyl alcohol, chloroform, and heat.

### Dynamic light scattering

**Fig. 10:**
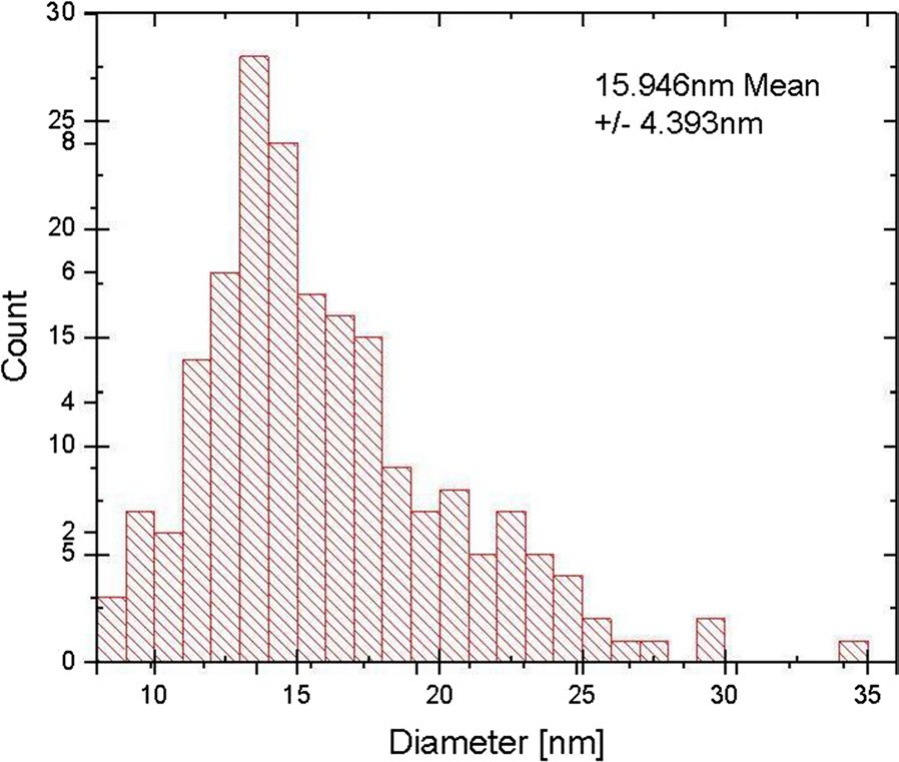
DLS size-distribution histogram of iron-oxide NPs prior to polymer coating

DLS results on OA-capped NPs right after synthesis returned an average diameter of ~ 16 nm (Fig. 10), in agreement with the TEM observations. PEG-5000 has a theoretical average length of ~ 30 nm, however it is important to note that the polymer length is just an average value, in addition the polymer chain can bend and twist resulting in a range of measured values. In our experiments, the succinylated PEG-5000 capping increased the hydrodynamic size of the NPs from 16 to ~ 41 nm.

**Fig. 11:**
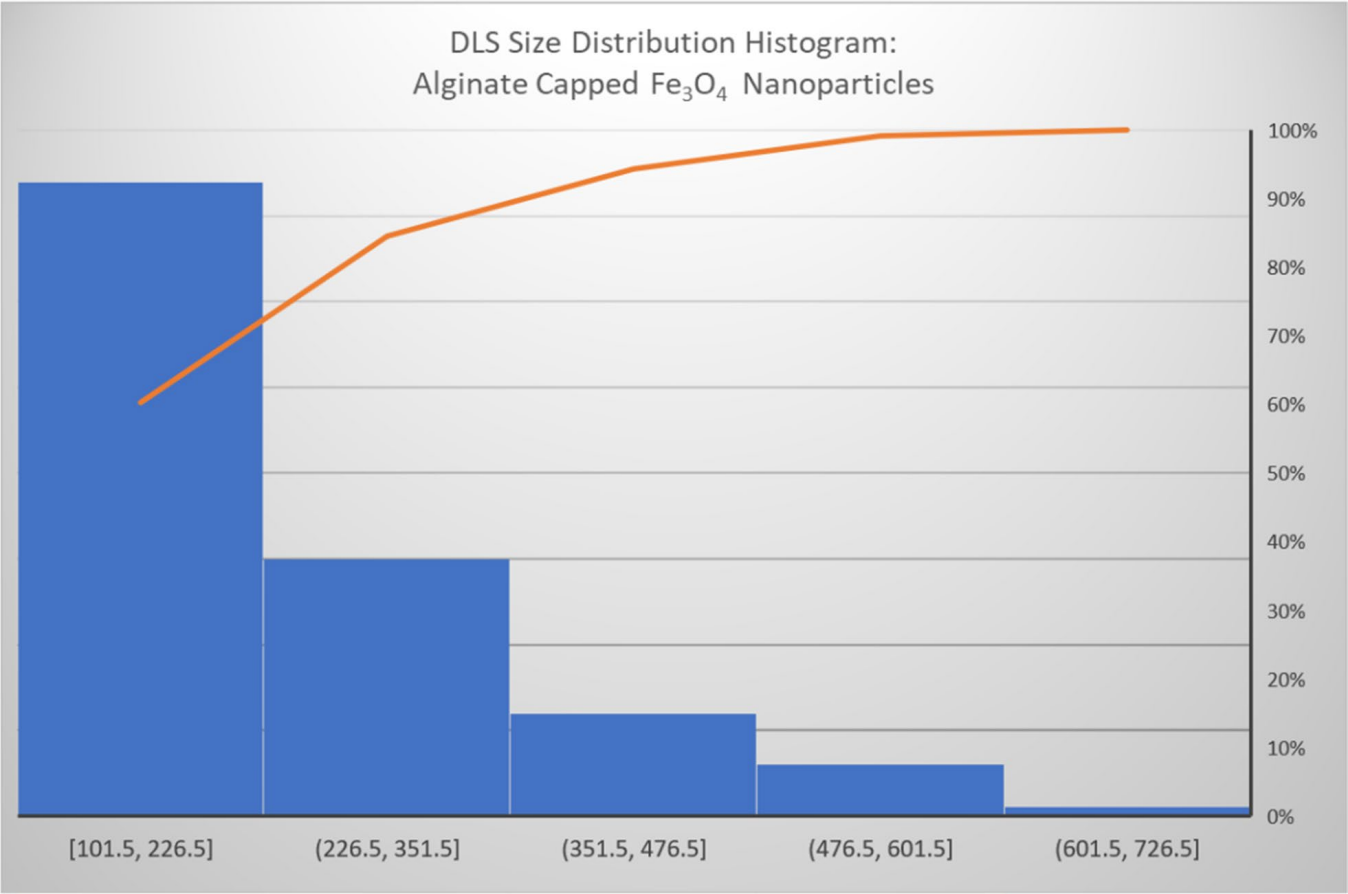
DLS size distribution showing average hydrodynamic size of iron-oxide NPs after alginate capping

Alginate capping, using the natural alginate, also having a range of polymer lengths, increased the hydrodynamic size of the NPs to 229.71 nm (Fig. 11). Tobramycin conjugation did not alter hydrodynamic size, as expected, due to the small sizes of both the tobramycin molecule and the crosslinker. Tobramycin conjugation was confirmed by FTIR spectroscopy.

Table 1 contains a summary of DLS results for all NPs used in this study.

**Table 1:** Summary of DLS results

Material	Hydrodynamic size
Iron-oxide:oleate NPs	16 nm ± 4.4 nm
Iron-oxide:PEG NPs	40.65 nm ± 0.83 nm
Iron-oxide:alginate NPs	229.71 nm
Iron-nitride:uncapped NPs	39.25 nm ± 21.40 nm
Iron-nitride:alginate NPs	267.6 ± 128.6 nm
Zero-valent iron:alginate NPs	241.571 ± 126.4 nm

#### Magnetic characterization

**Fig. 12:**
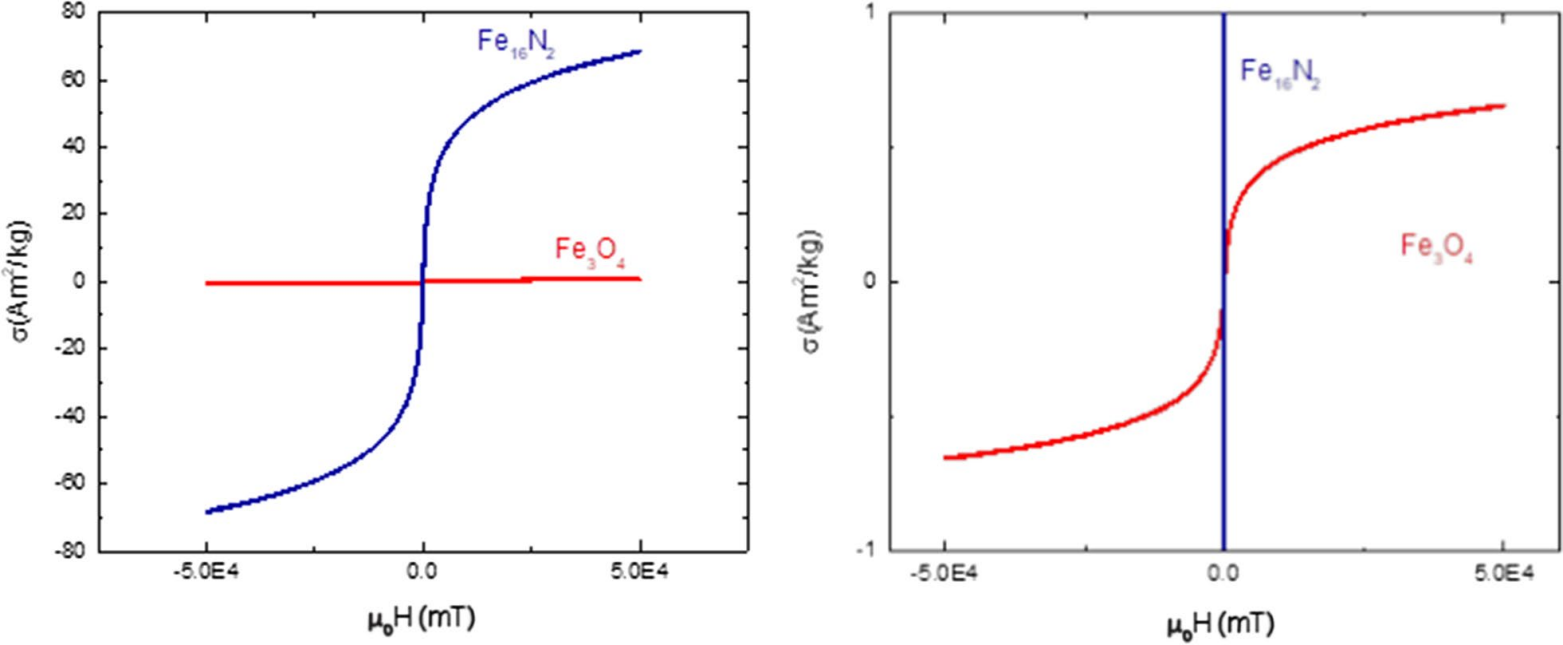
Comparison of hysteresis loops of nanocrystalline samples of iron oxide (red) and iron nitride (blue) of similar grain size, showing the significantly stronger magnetic properties of iron nitride. Left image shows entire hysteresis loop of iron nitride. Right image is a close up of the same, showing hysteresis loop of iron oxide

A typical feature in magnetic NPs is their irreversible ferromagnetic behavior below the blocking temperature T_B_ and reversible magnetization above it, caused by superparamagnetic behavior of the NPs. Below T_B_, the Néel relaxation time τ_N_ is larger than the measurement time τ_m_ (typically 100 s), and magnetization depends strongly on the field history. Above T_B_, magnetization is strongly affected by thermal fluctuations (τ_m_ > τ_N_), making FC and ZFC curves coincide. Therefore, for a given measurement time τ_m_, hysteretic behavior observed below T_B_ would not be observed above T_B_. The NPs displayed no hysteresis (no coercivity) under full magnetization *vs* field strength (M/H) sweep at 293 K (Fig. 12), a typical feature of superparamagnetic materials.

**Fig. 13:**
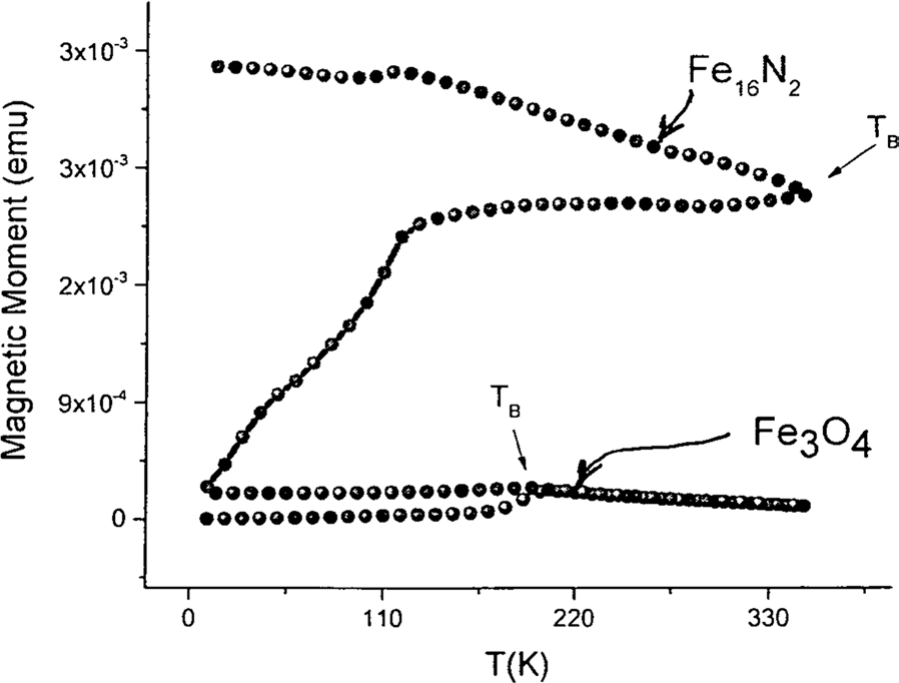
Magnetization vs temperature for iron-oxide and iron-nitride NPs under zero-field cooled (lower curves) and field cooled (upper curves) conditions. The magnetization of the ferrofluid samples was measured with a DC field of 100 Oe (τ_m_ = 100 s) in the temperature range between 9 K and 350 K

The DC (τ_m_ = 100 s) magnetization of the ferrofluid samples was measured with a DC field of 100 Oe in the temperature range between 9 and 350 K using a Quantum Design™ magnetic property measurement system (MPMS) superconducting quantum interference device (SQUID). In the entire temperature range up to 350 K, the Fe_16_N_2_ NP samples demonstrated strong ferromagnetic behavior, as evidenced by the gap between the ZFC and FC curves persisting even at 350 K. From the ZFC curve, we can loosely estimate T_B_ to be ~ 350 K, but even above that temperature equilibrium magnetization of the NP sample was not reached. Superparamagnetic behavior of the Fe_16_N_2_ NP samples was confirmed in magnetic hysteresis measurements. Consistent with the results of DC magnetization measurements, magnetic hysteresis measurements at 293 K performed on Fe_16_N_2_ NPs found no coercivity. We were unable to find M_sat_ with the field strengths presently available. Extrapolating the line gives a loose estimate of M_sat_ ~ 100 emu/g. The same measurement performed on the iron-oxide NP samples revealed a blocking temperature of approximately 150 K, far below the measured value for the iron nitride sample (Fig. 13).

#### Zeta potential results

Table 2 summarizes the zeta potential measurements for the listed samples. The best results have been obtained for the NPs coated with alginate. It is possible that even higher colloidal stability could be achieved with a thicker alginate coating, leading to a more negative zeta potential. However, it is important to note that a larger sized particles may have more difficulty diffusing through the pores in the mucus/biofilms. It appears that sufficient charge shielding, and acceptable colloidal stability has been achieved and is balanced with the desired hydrodynamic size (~ 250 nm).

**Table 2:** Summary of zeta potential results

Material	Solvent	Zeta potential (mV)	Standard deviation (mV)
Iron-oxide:oleic acid NPs	Chloroform	− 11.1	7.46
Iron-oxide:PEG NPs	DI water	− 4.23	6.92
Iron-oxide:alginate NPs	DI water	− 31.5	6.33
Iron-nitride:alginate NPs	DI water	− 25.2	7.63
Zero-valent iron:alginate NPs	DI water	− 22.1	5.8
Iron oxide:alginate:tobra NPs	DI water	− 15.1	7.18

#### FTIR and confirmation of tobramycin conjugation

The presence of an amide stretch, visible on FTIR at 1630–1680 cm^−1^ was used to verify the success of the crosslinking procedure. Loading efficiency of tobramycin calculated as mass of NP conjugates/mass of alginate-capped NPs was found to be ~ 2%.

### Motility testing results

#### Motility testing

**Fig. 14:**
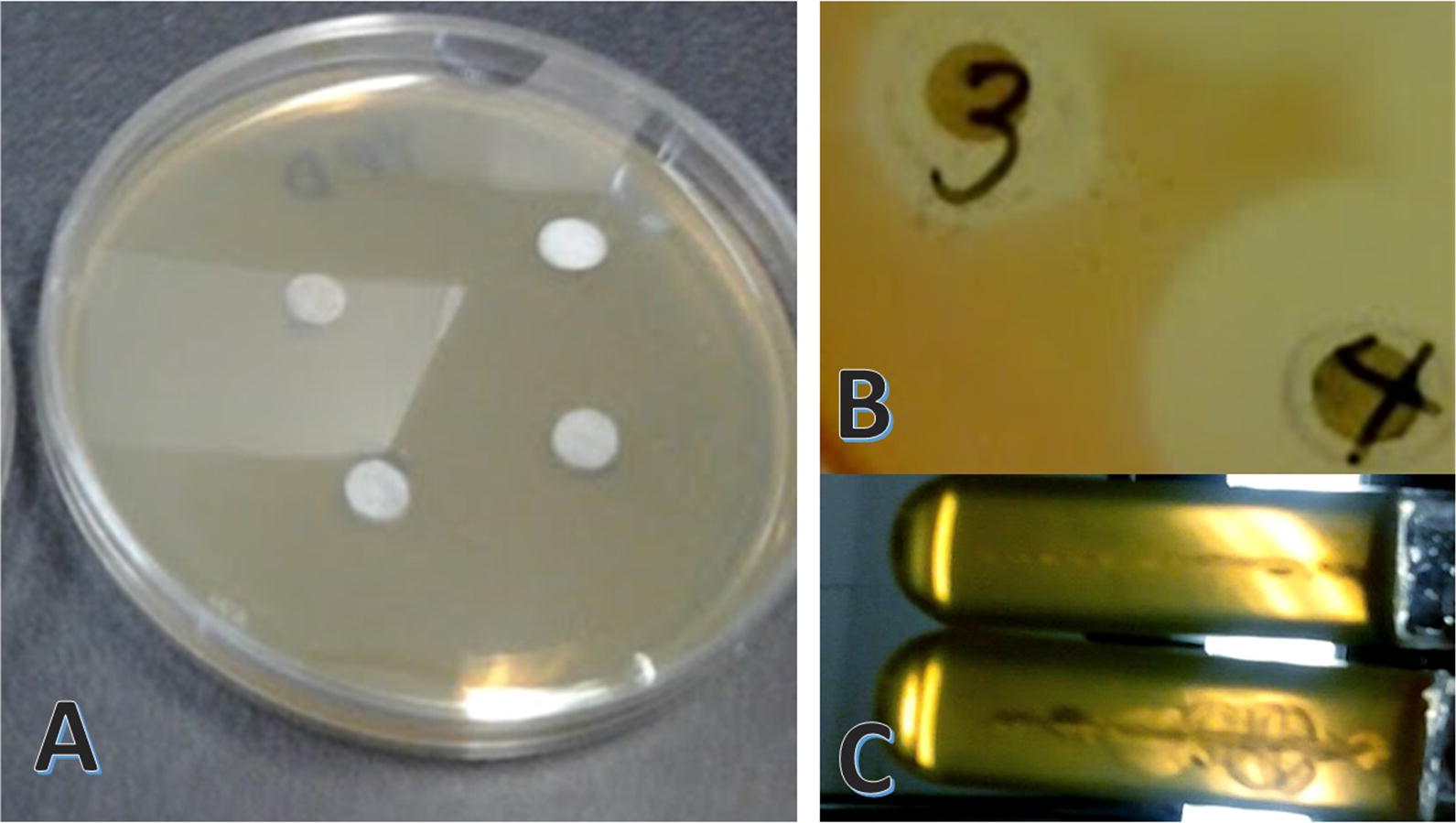
Agar cultures used for susceptibility testing. A) Agar plate with impregnated disks prior to overnight incubation. B) Image shows zone of inhibition (ZOI) halo around disk impregnated with antimicrobial agent of interest; a positive susceptibility result. C) Motility testing results in agar stab cultures after incubation; upper tube is a negative motility result and lower tube is a positive motility result

*P. aeruginosa* was not found to be motile after an overnight incubation in liquid growth media. Cultures taken from biofilms were found to exhibit motility after 3-days of growth (see image results in Fig. 14). These findings attest to the increase in genetic diversity in the established biofilms.

### Susceptibility results determined via disk diffusion method

Figure 14 shows the disk diffusion experiment before and after incubation and the motility testing results, all performed using solid agar. Disk diffusion results for tobramycin were interpreted based on the 2019 CLSI breakpoints for tobramycin in *P. aeruginosa* [178], in which the mass of tobramycin on the disk is 10 µg and a disk diameter of ≥ 15 mm is susceptible (S), 13–14 mm is intermediate (I) and ≤ 12 mm is resistant (R). Since there exist no standards for the investigation of iron oxide nanoparticle susceptibility in any microbes, we used the same cutoff values as for tobramycin in order to maintain consistency. We also investigated a range of concentrations of tobramycin, NPs, and NP-conjugates in order to determine susceptibility range. Over time, the disk diffusion results (Table 3), taken together with the MIC results, demonstrate that the tobramycin susceptibility decreases and resistance increases as the colonies are allowed to grow in biofilm mode for longer periods of time, despite being tobramycin naïve. Therefore, this is not due to exposure-related resistance development. It is important to note that the observed increase in resistance is not due to a larger initial amount of CFU’s in the 60-day old biofilms, because cultures were diluted and identical concentrations of CFUs were used for inoculation and plating for all time periods. These findings suggest that the age of the infection alone (*i.e*., establishment of a chronic infection) contributes to resistance. This is possibly due to broader genetic diversity in the population. No comparable increase in resistance over time was observed for the NP samples investigated, suggesting that a genetic resistance mechanism to counter the action of the compound may not exist. We can speculate that the mechanism of action of the iron-oxide NPs is not based on inhibition of genes or bacterial protein synthesis, which implies the toxicity may not be prokaryote-specific.

**Table 3:** Susceptibility of *P. aeruginosa* biofilms to various treatments after 3 and 60 days of growth, determined by disk diffusion

Material	Dose on disk (μg)	ZOI (mm) day 3	ZOI (mm) day 60
Fe_3_O_4_ NPs^†^	10	22/S	21/S
	5	17.5/S	16/S
	2.5	11/R	10/R
Fe_3_O_4_@PEG NPs	10	0/R	0/R
	5	0/R	0/R
	2.5	0/R	0/R
Fe_3_O_4_@ALG NPs	10	22/S	22/S
	5	16/S	15/S
	2.5	10/R	8/R
Fe_3_O_4_@ALG:TOBRA NPs	10	23/S	22/S
	5	11/R	15/I
	2.5	7/R	5/R
ZVFe@ALG NPs	10	25/S	24/S
	5	21/S	22/S
	2.5	20/S	20/S
Tobramycin	10*	10/R	0/R
	100**	25/R**	15/R**
	1000**	35/R**	32/R**

Interpretation: *R* resistant, *I* bintermediate, *S* susceptible, *ZOI* zone of inhibition, *PEG* polyethylene glycol, *ALG* alginate, *TOBRA* tobramycin, *ZVFe* zero-valent iron

^†^Uncapped NPs. *CLSI breakpoint for susceptibility of tobramycin by disk diffusion is 10 μg. Therefore, all colonies are found to be tobramycin resistant by CLSI standards. **Higher tobramycin doses in the disk were investigated to determine whether any susceptibility existed at higher doses. At present, there are no CLSI values/breakpoints for NPs as antimicrobial agents

For the iron-oxide NPs alone, we found that inhibition of established biofilms on agar plates was observed for low concentrations. When capped with alginate, the inhibition remained low even though part of the mass of this core–shell type NP consists of non-bioactive alginate. In the case of iron-oxide NPs capped with PEG, no inhibition was observed, possibly because the non-biodegradable nature of the capping agent may keep the iron from interacting directly with the bacteria (see Table 3). If the iron ions contribute to the toxicity, it may be possible that, in this case, they were not distributed to the colonies, and therefore, could not inhibit bacterial growth.

Regardless of the mechanism, which has yet to be established, these findings demonstrate that the crucial role of the capping agent to the impartation of antimicrobial properties. Therefore, the capping agent also contributes to or negates the toxicity of this material. We can speculate that a complete PEG cap may also reduce the toxicity of NPs known to exhibit cytotoxic effects in vivo, since it appears to limit interaction with the cells, at least in this short exposure time frame.

Even at high concentrations, we might expect to observe some inhibition due to incomplete coverage, however, that is not the case. In the case of iron-oxide NPs conjugated to tobramycin, we find that the bacterial inhibition at these concentrations mirrors the inhibition trend of iron-oxide NPs alone. It is important to note that these findings are characteristic of this particular strain, after this period of growth, and its susceptibility to tobramycin. One previous study found that after a 1000 μg/mL concentration of tobramycin was applied to established biofilms, a significant proportion of the bacterial cells were still viable after 12 h [181]. This group also reported that planktonic cells taken from the same strain was completely killed by only 50 μg/mL. Another relevant study reports the MIC from their clinical isolates to be 8 μg/mL [130]. These published findings suggest a huge theoretical therapeutic dose ranging from 8 to more than 1000 μg/mL. MIC and susceptibilities appear to differ dramatically from strain to strain and in planktonic vs biofilm communities. Therefore, it is probable that these susceptibilities may also differ from strain to strain and under different growth conditions.

### Inhibitory concentration of tobramycin

**Fig. 15:**
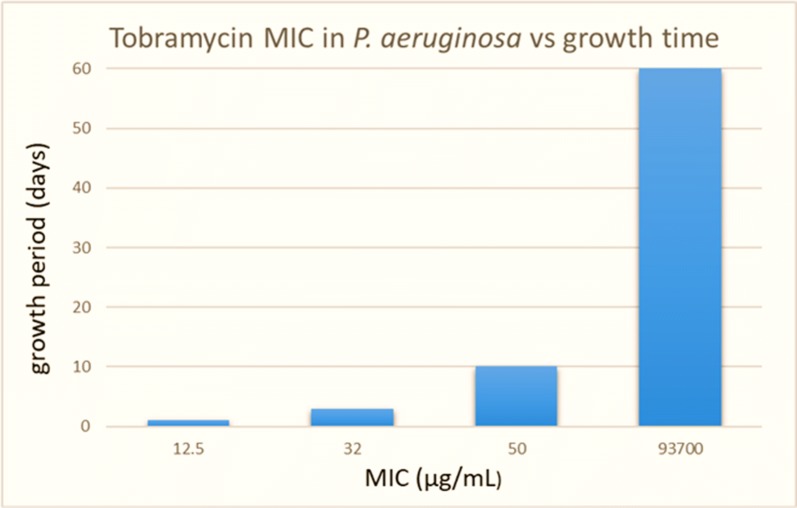
Minimum inhibitory concentration (MIC) of tobramycin on *P. aeruginosa* colonies as a function of growth time. Note that the cutoff concentration for susceptibility of *P. aeruginosa* to tobramycin in liquid cultures is ≤ 4 μg/mL. Therefore, none of the cultures are susceptible to tobramycin by CLSI standards

The MIC of tobramycin in this strain of *P. aeruginosa* was determined for several time points in liquid and biofilm cultures, and found to be 10–15 μg/mL for planktonic cells grown overnight in liquid broth, 32 μg/mL for 3-day old biofilm cells, 50 µg/mL for 10-day old biofilms and 93.7 mg/mL for 60-day old biofilms (Fig. 15).

All biofilm colonies were maintained in liquid broth on sterile boiling stones, having the broth was replaced daily. The MIC of tobramycin differs significantly from strain to strain and when comparing planktonic vs biofilm cells, as well as biofilm growth time. These trends were not observed for shorter periods of growth. These findings add merit to our longer-term growth period for the establishment of biofilm colonies. According to the breakpoints recommended by the CLSI for determination of MIC; inhibition at a concentration ≤ 4 µg/mL of tobramycin means the strain is susceptible; inhibition at a concentration of 8 µg/mL is intermediate; and inhibition at concentrations ≥ 16 µg/mL means the strain is tobramycin resistant [178]. Therefore, according to the CLSI breakpoints for interpretation of MIC, these cultures were never found to be susceptible to tobramycin. The biofilm cultures were found to be tobramycin-resistant and becoming more resistant over time.

### CF mucin alginate barrier model

The CF model, in which artificial mucin and alginate barriers were applied over the agar-grown colonies cultured from established 60-day old biofilms, reveals that application of an external magnetic field enhances susceptibility to the iron-oxide NPs and NP-drug conjugates, possibly by promoting transport across the two barriers. For this study, 50 mg/mL concentrations of NPs and NP-conjugate solution were applied to the disk, such that each disk contained 50 µg of test article. The results without (Table 4) and with (Table 5) magnetic field application demonstrate zero susceptibility to tobramycin alone. No CLSI breakpoints exist for NPs or NP conjugates at present, however, the CLSI cutoff values for determination of *P. aeruginosa* susceptibility to tobramycin with 10 μg absorbed onto a disk are ≥ 15 susceptible, 13–14 intermediate, and ≤ 12 resistant [178]. The same parameters were used for interpretation of the NP and NP-conjugate results. Tables 4 and 5 demonstrate the highly statistically significant contribution of the external magnetic field in enhancing susceptibility to the test articles. More work is needed to determine the exact role of the magnetic field in addition to determining the minimum or maximum field strength necessary to achieve maximum susceptibility. It is possible that the pulling force of the magnet may relate in some way to the thickness of the biofilm and mucus barriers.

**Table 4:** Susceptibility of *P. aeruginosa* colonies beneath mucin, alginate, or alginate and mucin barriers, determined by disk diffusion

Disk no./compound	Mucin	Alginate	Alginate + Mucin
1. Fe_3_O_4_ NPs† 10 μg	14/I	0/R	22/S
2. Fe_3_O_4_@ALG:TOBRA 10 μg	0/R	0/R	0/R
3. Tobramycin 10 μg	0/R	0/R	0/R
4. Tobramycin 100 μg**	11/R**	14/R**	5/R**
5. Tobramycin 200 mg**	30/R**	35/R**	30/R**

Disk diffusion method was used. Minimum concentrations demonstrating susceptibility in previous disk diffusion studies were used for NPs and NP-tobramycin conjugates. ^†^Uncapped NPs, ALG: alginate, TOBRA: tobramycin

* Maximum CLSI cutoff concentration for susceptibility of tobramycin 10 μg absorbed onto disk

** Corresponding to experimentally determined susceptibility, these doses of tobramycin shown were up to five orders of magnitude higher than the CLSI standard dose for disk diffusion. Therefore, although inhibition was observed, these colonies were tobramycin resistant by CLSI standard

**Table 5:** Susceptibility of *P. aeruginosa* colonies beneath mucin, alginate, or alginate and mucin barriers with external magnetic field application

Disk no. compound	Mucin barrier	Alginate barrier	Mucin + alginate barriers
1. Fe_3_O_4_ NPs† 10 μg	30/I	0/R	20/S
2. Fe_3_O_4_ @ALG:TOBRA	25/R	19/R	14/R
3. Tobramycin*	0 R	0/R	0/R
4. Tobramycin 100 μg**	10/R**	12/R**	0/R**
5. Tobramycin 200 mg**	32/R**	30/R**	20/R**

Disk diffusion method was used. Minimum concentrations demonstrating susceptibility in previous disk diffusion studies were used for NPs and NP-tobramycin conjugates. †Uncapped NPs, *ALG* alginate, *TOBRA* tobramycin

* Maximum CLSI cutoff concentration for susceptibility of tobramycin 10 μg absorbed onto disk

** Corresponding to experimentally determined susceptibility, these doses of tobramycin were up to five orders of magnitude higher than the CLSI standard dose for disk diffusion. Therefore, these colonies were tobramycin resistant by CLSI standard

### Biofilm inhibition in liquid cultures

**Fig. 16:**
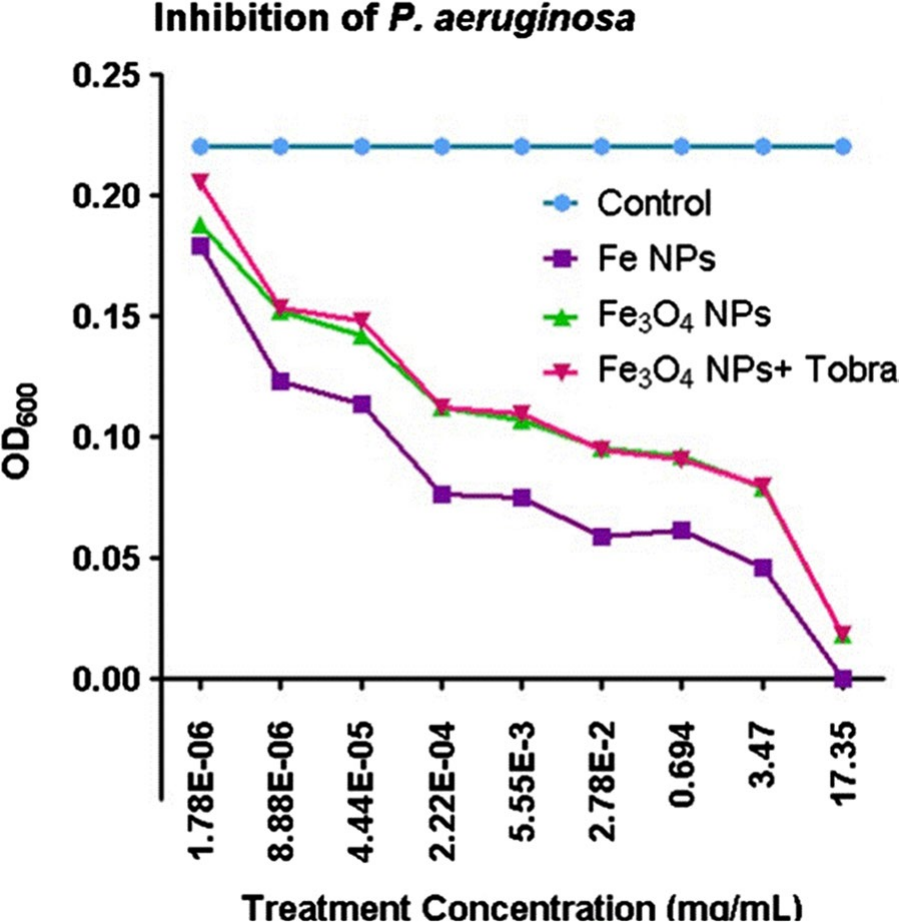
Optical density (OD) at a 600 nm wavelength for liquid cultures exposed to treatment with iron-oxide NPs, zero-valent iron, or tobramycin-conjugated iron-oxide NPs. To avoid interference, NPs were removed by magnetic separation prior to optical measurements. The calculated average error for OD measurements was ± 0.01. Specific errors, not the average error, were used to calculate statistical significance

Biofilm inhibition and MIC of liquid cultures was determined by taking a standardized aliquot from established 60-day old biofilm communities, inoculating a standardized volume of liquid broth containing the desired amount of drug or broth only (for the negative control), and then growing for 24-h. OD_600_ was determined against a blank cuvette, containing broth only. The OD_600_ of the negative control samples (containing only inoculated broth) was determined to be 0.22 to 0.24. This result is slightly higher than the lowest treatment concentration (8 × 10^−6^ mg/mL). Due to the lack of prior art suggesting an inhibitory concentration, it was necessary to investigate a large range of concentrations to determine MIC. The range used was 17.35 mg/mL to 8 × 10^−6^ mg/mL, in a consistent volume, determined by serial dilution, as the graph in Fig. 16 illustrates. Complete inhibition was observed for all materials at concentrations at 17.5 mg/mL (or higher), and various degrees of inhibition fall off somewhat linearly at concentrations below 17.35 mg/mL (Fig. 16). The inhibition by zero-valent iron was, not surprisingly, higher than iron-oxide NPs and NP-drug conjugates. We attribute this to the high reactivity of zero-valent iron and its ability to increase reactive oxygen species (ROS) in the local region [182]. Although speculative at this stage, it is also possible that high levels of iron contribute to cellular toxicity. More work is necessary to determine toxic and non-toxic dose ranges.

**Fig. 17:**
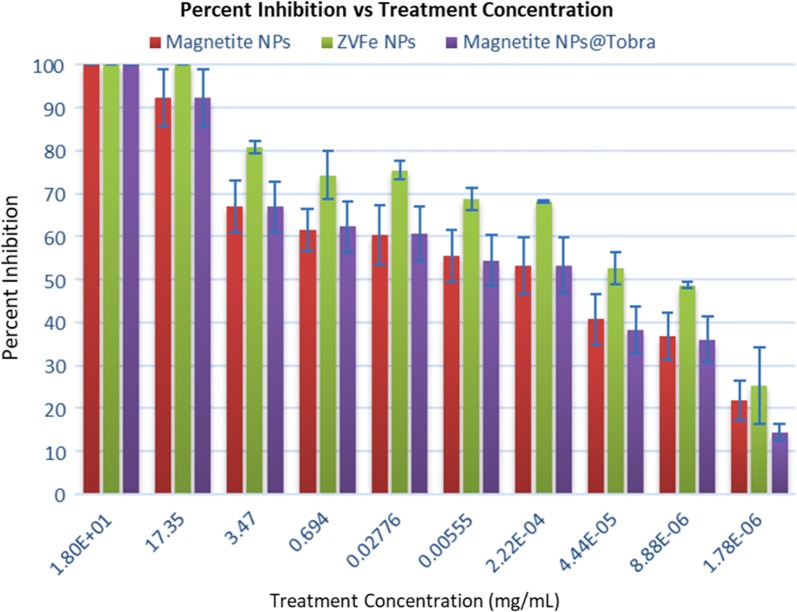
Percent bacterial inhibition vs. treatment concentration in liquid cultures in cuvette. All NP samples presented here were capped with alginate

ANOVA results showed that, while there was no statistically significant difference between the zero-valent iron, iron oxide, or iron-oxide—tobramycin conjugates; when compared to control, the results for all three NP treatments were found to be extremely statistically significant (*p* < 0.0001). Figure 17 shows that the inhibition of bacterial cells was evident even at surprisingly low (8 ng/mL) concentrations, although the minimum therapeutic dose would probably be much higher; the range in which more significant bacterial inhibition was observed. Speculation on a therapeutic dose for targeted delivery would likely differ from the systemic dose, and both will depend on observed cytotoxicity in mammalian cell cultures, at these concentrations. Even higher doses may be required for the treatment of chronic infections involving biofilms that have been established for several years; however, more research is necessary to determine this.

These findings attest to the feasibility using these materials as antimicrobial coatings or therapeutic agents. The MIC for different strains of *P. aeruginosa* may differ as well. According to another report, *P. aeruginosa* (MTTC 1034) was not found to be susceptible to iron-oxide NPs at 50 mg/mL, whereas our strain exhibited positive susceptibility [120]. It has been shown previously that oxygen limitation and metabolic activity can alter MIC of tobramycin in *P. aeruginosa* [183]. Differences in zone diameter for susceptibility testing have also been known to differ with different batches of growth agar [184]. [118] reported positive bacterial inhibition for *P. aeruginosa* PAO1, in agreement with our findings. We attribute differences in susceptibilities to genetic differences among strains in combination with the contribution of environmental factors, such as growth media and the use of different capping agents.

The mechanism by which iron-oxide NPs exhibit antibacterial activity remains unknown. However, according to the findings of [185], iron may very well be the bioactive component. Zero-valent iron, as predicted, had a dramatic antibacterial effect, verifying the findings of [186]. Although zero-valent iron is too reactive for in vivo use at present, it may be a candidate for incorporation into antibacterial coatings. Similarly, iron-oxide NPs having high biocompatibility, may be a candidate material for incorporation into polymer for use as antibacterial coatings on virtually any inert surface used outside of the body, as well as medical devices such as stents, catheters, and surgical sutures as a low-cost alternative to silver NPs. We anticipate that the combination of tobramycin or other drugs with iron-oxide NPs incorporated into biodegradable polymers may hold promise for the long-term control of biofilms and multidrug resistant microbial strains. More work is needed to determine antibacterial properties of these materials on other microbial species.

## Discussion

Although we have demonstrated the antibacterial properties of NP-drug conjugates or NPs alone, it is not yet known if the NPs or NP-drug conjugates cross the bacterial cell membrane, or if action uptake by the cell is not necessary for the antibacterial effect. It has been shown that aminoglycoside antibiotics, such as tobramycin, enter the cell through porin channels, as altered permeability of the cellular membrane is known to lead to antibiotic resistance [187–189]. *P. aeruginosa* has previously been shown to possess a relatively large exclusion limit having a ~ 3 kDa cutoff [190].

The exact mechanism of the antimicrobial action of Fe_3_O_4_ NPs that results in damaging the bacterial proteins and DNA has not yet been determined, but it has been hypothesized that it might involve oxidative stress caused by reactive oxygen species, such as superoxide radicals (O_2_^−^), singlet oxygen (^1^O_2_), hydroxyl radicals (–OH), or hydrogen peroxide (H_2_O_2_) [191]. For example, H_2_O_2_ could penetrate the cell membrane of the bacteria and kill the bacteria by entering the intracellular space. It appears that a destructive consequence appears under of greater concentrations of zero-valent iron and consequently, ROS induced by the presence of iron. Experimentation to determine the bactericidal effects of zero-valent iron and the theoretical mechanisms leading to cell death has been done previously, and the established findings may be referenced in any of the following notable publications. The first of which reports significant disruption of the *Escherichia coli* cell membrane by zero-valent iron NPs suggesting inactivation or enhanced the biocidal effects of dissolved iron as well as oxidative stress as mechanisms of cell death [192]. Another report, [193], investigated the use of zero-valent iron NPs against Gram negative *Escherichia coli* and Gram-positive *Bacillus subtilis* showing that *B. subtilis* was more tolerant to zero-valent iron NPs than *E. coli,* but states that the bactericidal mechanism has not yet been elucidated. Despite these findings, there is not yet a consensus in the literature regarding this effect; another paper published elsewhere claims that zero-valent iron had no deleterious effect on total bacterial abundance in the microcosms. This paper reports that zero-valent iron with a biodegradable polyaspartate cap increased bacterial populations by an order of magnitude relative to controls [194]. Perhaps, upon reaching stable oxidation equilibrium in the body, this material will benefit bacterial populations by providing beneficial doses of iron.

It is possible that iron NPs may indirectly generate ROS, which subsequently damage iron–sulfur clusters located in an assortment of metalloproteins, examples are the well-known nicotinamide adenine dinucleotide (NADH) dehydrogenase, ferredoxins, hydrogenases, nitrogenase, coenzyme Q, and succinate dehydrogenase [195]. The combination of radicals and aqueous iron produces Fenton’s reagent. Fenton’s reagent is a solution of hydrogen peroxide and iron. In industrial applications, Fenton’s reagent can be used to destroy organic compounds by catalyzing the production of additional ROS. ROS generated by this reaction can easily diffuse into the cell cytoplasm, triggering ROS-induced release in the mitochondria triggering death.

Another way that the Fenton’s reagent mechanism may be applicable to the antibacterial effects of iron oxide is free-radical DNA damage. The superoxide anion ($O_{2}^{ \cdot - }$) can dismutate to form hydrogen peroxide H_2_O_2_ [196]. $O_{2}^{ \cdot - }$ can also reduce Fe(III), thus releasing it from the ferritin protein according to:

$O_{2}^{ \cdot - } + Fe3^{ + } \to Fe^{2 + } + O_{2}$, free iron(II) from iron-sulfur clusters according to: $\left[ {4Fe - S]^{2 + } + O_{2}^{ \cdot - } + 2H^{ + } \to } \right[3Fe - 4S]^{ + } + H_{2} O_{2} + Fe^{2 + }$, and subsequently, very reactive oxygen species can form through the Fenton reaction $Fe^{2 + } + H^{ + } + H_{2} O_{2} \to Fe^{3 + } + \cdot OH + H_{2} O_{2}$.

**Fig. 18:**
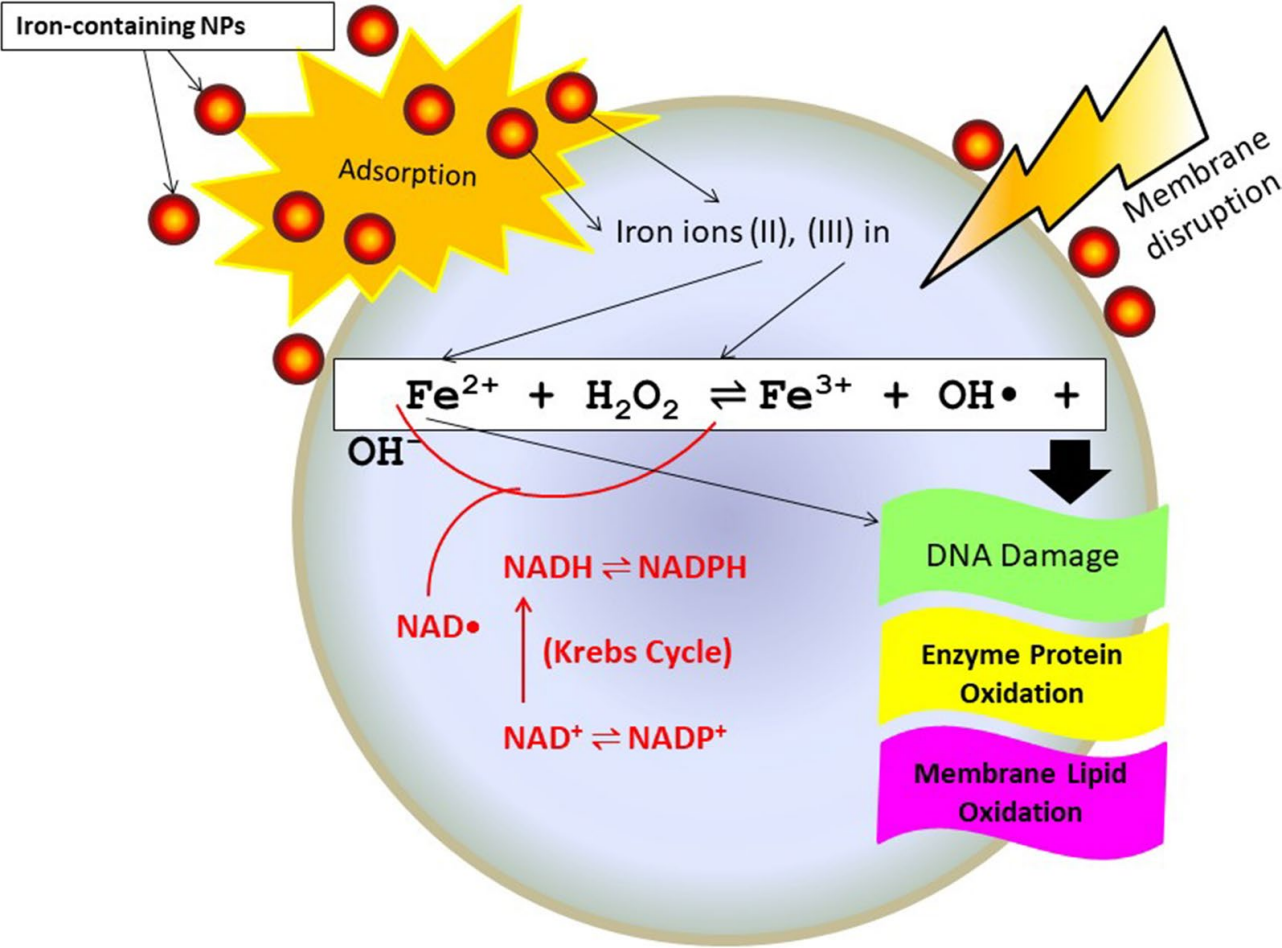
Mechanisms of cell damage and response after exposure to iron-containing NPs. Iron ions, released from NPs, can cross the membrane via either active cellular uptake or leakage through sites with reduced membrane integrity. Highly reactive hydroxyl radicals resulting from Fe^2+^ reaction with hydrogen peroxide primarily cause oxidative damage. Fe^3+^ could be reduced by NADH and, thus, regenerating Fe^2+^. OH·radicals could also cause damage to DNA, proteins and lipids. Fe^2+^ may also directly damage DNA

The Fenton reaction has been linked to DNA damage by its products: iron, O_2_, and H_2_O_2_ [196]. Fe^3+^ produced by the Fenton reaction is thought to be reduced by available NADH, thus replenishing Fe^2+^, H_2_O_2_ can be depleted by catalase or by peroxidases, which utilize reduced glutathione, other thiols, cytochrome *c*, and ascorbate [196]. Mechanisms of bacterial cell death induced by the Fenton reaction are illustrated in Fig. 18.

It may also be possible that initial disruption of the outside membrane of bacteria by tobramycin assists the subsequent penetration of NP-tobramycin complexes and or iron ions into the bacterial cell via simple diffusion since it is known that one mechanism of action of aminoglycoside antibiotics is interference with protein synthesis leading to cell membrane disruption. However, if this is occurring, it is not happening on a large scale since no statistically significant difference in the MIC or susceptibility was noted in tobramycin conjugated iron-oxide NPs compared to unconjugated NPs. However, more work is needed to clarify the antibacterial mechanism(s) of action of iron-oxide NPs alone and in combination with the aminoglycoside, or other antibiotic drugs, and to clarify the overall role of the capping agent.

It is apparent that the composition of the capping agent, and possibly the interactions of the capping agent with the NP surface, the ROS, and the cell surfaces are primarily responsible for facilitating or negating the antimicrobial effects. Since uncapped iron-oxide NPs (~ 16 nm) had similar antibacterial effects as the alginate-capped and alginate-capped/tobramycin conjugated NPs (~ 200 nm), whereas the PEG-capped NPs (~ 40 nm) were ineffective, we do not attribute these findings to size effects. At least at this size range.

## Conclusions

We report the susceptibility and inhibitory concentrations of iron-oxide (nominally magnetite) nanoparticles (NPs) with and without attached drug (tobramycin) against *P. aeruginosa* PAO1 communities. The NPs investigated in this study have an average hydrodynamic diameter of 16 nm, prior to capping with natural alginate. The NP-drug conjugates were prepared using 1-ethyl-3-(3-dimethylaminopropyl)carbodiimide hydrochloride/N-hydroxysulfosuccinimide (EDC/sulfo-NHS) crosslinking of the aminoglycoside antibiotic tobramycin with alginate, by way of carboxyl and amine functional groups. Bacterial biofilm cultures were grown for 60 days to more closely model an established infection. We also investigated an in vitro CF model, in which a magnetic gradient was applied to NP-drug conjugates atop an artificial biofilm (alginate layer) and mucus layer on agar. Positive inhibition of bacterial growth was observed for uncapped and alginate-capped iron-oxide NPs, and the corresponding MICs have been presented. We have observed zero susceptibility to iron-oxide NPs capped with PEG, suggesting that the capping agent plays a major role in enabling bactericidal ability in of the nanocomposite. Our findings suggest that the alginate-coated nanocomposites investigated in this study have the potential to overcome the bacterial biofilm barrier, possibly by simple diffusion, due to the favorable solubility of the alginate-coated NPs within the alginate biofilm. Magnetic field application increases the action, likely via enhanced diffusion of the iron-oxide NPs and NP-drug conjugates through mucin and alginate barriers, which are characteristic of CF respiratory infections. We have demonstrated that iron-oxide NPs coated with alginate, as well as alginate-coated magnetite—tobramycin conjugates inhibit *P. aeruginosa* growth and biofilm formation in established colonies, which are often the most difficult to treat. We have also determined that susceptibility to tobramycin decreases for longer culture times, as the colonies are allowed to differentiate for longer periods of time. In contrast, susceptibility to the iron-oxide NP compounds did not demonstrate any comparable decrease with increasing culture time. These findings appear to imply that iron-oxide NPs are promising lower-cost alternatives to silver NPs in antibacterial coatings, solutions, and drugs, as well as other applications in which microbial abolition or infestation prevention is sought. Future work should include multiple strains of *P. aeruginosa* in order to determine to what extend the response may be strain-dependent, as well as detailed investigations into the mechanism(s) of action.

## Data Availability

All data generated or analyzed, and materials used in this study are included in this manuscript.

## Abbreviations

NP: Nanoparticle
CF: Cystic fibrosis
MDR: Multidrug resistant
ICU: Intensive care unit
VAP: Ventilator-associated pneumonia
EPS: Extracellular polysaccharide
M: Mannuronic acid
G: Guluronic acid
PEG: Polyethylene glycol
EDC: 1-ethyl-3-(3-dimethylaminopropyl)carbodiimide
TEM: Transmission electron microscopy
DLS: Dynamic light scattering
XRD: X-ray diffraction
EDS: Energy-dispersive X-ray spectroscopy
MIC: Minimum inhibitory concentration
DI: Deionized
mPEG: Methyl-terminated PEG
NHS: N-hydroxysuccinimide
FTIR: Fourier-transform infrared
HRTEM: High-resolution TEM
MPMS: Magnetic property measurement system
SQUID: Superconducting quantum interference device
ZFC: Zero-field cooling
FC: Field cooling
UV–VIS–IR: Ultraviolet–visible–infrared
OD: Optical density
LB: Luria–Bertani
CFU: Colony forming unit
ZOI: Zone of inhibition
CLSI: Clinical and Laboratory Standards Institute
ROS: Reactive oxygen species
NADH: Nicotinamide adenine dinucleotide
